# Fear of depression recurrence among individuals with remitted depression: a qualitative interview study

**DOI:** 10.1186/s12888-024-05588-4

**Published:** 2024-02-21

**Authors:** Stephanie T. Gumuchian, Ariel Boyle, Lori H. Hazel, Mark A. Ellenbogen

**Affiliations:** Department of Psychology, Concordia University, 7141 Sherbrooke Street West, H4B 1R6 Montréal, Québec Canada

**Keywords:** Major depressive disorder, Fear of depression recurrence, Qualitative inquiry, Content analysis

## Abstract

**Background:**

Major Depressive Disorder (MDD) is a prevalent psychiatric condition and the largest contributor to disability worldwide. MDD is highly recurrent, yet little is known about the mechanisms that occur following a Major Depressive Episode (MDE) and underlie recurrence. We explored the concept of fear of depression recurrence (FoDR) and its impact on daily functioning among individuals in remission from MDD.

**Methods:**

30 participants (83% female; 37% White; M_age_ = 27.7, SD = 8.96) underwent semi-structured qualitative interviews. The interviews explored participants’ experiences of FoDR including the frequency, severity, content, triggers, and impact of fears and associated coping strategies. We used content analysis to analyze the transcriptions.

**Results:**

Most participants (73%) reported having FoDR, with varying frequency, severity, and duration of fears. The triggers and content of participants’ fears often mirrored the symptoms (e.g., low mood, anhedonia) and consequences (e.g., job loss, social withdrawal) endured during past MDEs. Some participants reported a minimal impact of FoDR on daily functioning, whereas others reported a positive (e.g., personal growth) or negative (e.g., increased anxiety) influence.

**Limitations:**

Our sample size did not allow for explorations of differences in FoDR across unique MDD subtypes or sociocultural factors.

**Conclusions:**

The concept of FoDR may present a window into understanding the unique cognitive and behavioural changes that occur following MDD remission and underlie depression recurrence. Future research should aim to identify underlying individual differences and characteristics of the disorder that may influence the presence and impact of FoDR. Finally, a FoDR measure should be developed so that associations between FoDR and recurrence risk, depressive symptoms, and other indices of functioning can be determined.

**Supplementary Information:**

The online version contains supplementary material available at 10.1186/s12888-024-05588-4.

## Background

Major Depressive Disorder (MDD) is a debilitating psychiatric condition and the largest contributor to disability worldwide [1]. It is considered to have the highest lifetime prevalence among psychiatric conditions, affecting over 300 million people [2]. MDD is considered a chronic condition as approximately 50–85% of individuals who have had at least one Major Depressive Episode (MDE) experience a second, with this percentage increasing for individuals with multiple past MDEs [3–5]. Despite mounting evidence of MDD’s high recurrence, little is known about the predictors and mechanisms underlying recurrence.

There are several limitations to research in MDD that challenge our ability to discern the conditions that lead to subsequent MDEs. These concerns include overreliance on cross-sectional methodologies, a lack of consensus on what constitutes recurrence, and grouping all individuals with MDD together instead of differentiating subgroups using key characteristics of the disorder (e.g., single verse recurrent episodes) [3, 6–8]. The existing literature focuses on identifying individual factors (e.g., genetic vulnerability) and evaluating the role of treatment in predicting future MDEs [3, 9]. Research on recurrence risk factors have identified that residual symptoms, anxiety disorders, childhood maltreatment, and previous MDEs are some of the strongest prognostic factors in recurrent depression [10]. A review exploring prospective biomarkers (e.g., hormones, oxidative stress) in MDD recurrence found that cortisol significantly increased odds for MDD onset and relapse [6]. These indicators, however, do not provide insight into the cognitive and behavioral changes that occur following MDE remission and underlie future recurrence [7].

Individuals may behave differently following an MDE out of fear of future relapse, such as reducing risk-taking and being hypervigilant to symptom changes [11]. Fear of illness recurrence (FIR) is defined as concern, fear, or worry that one’s illness will eventually return [12]. FIR has been widely studied in cancer and other chronic health conditions and is associated with greater avoidance of illness reminders (e.g., medical appointments), disregarding symptom changes, and social withdrawal [13–16]. FIR in cancer is associated with lower mood, greater depression and anxiety, reduced quality of life, and lower engagement in health behaviours [17–20].

Research on FIR in psychiatric conditions is scarce. Studies of psychotic disorders have reported that FIR significantly predicted future relapse and was associated with increased positive psychotic symptoms, depression, anxiety, and greater use of maladaptive coping strategies (e.g., reassurance seeking) [21, 22]. In MDD, some individuals have endorsed FIR following the discontinuation of antidepressants [23–25]. Others have reported that the fear surrounding experiencing another MDE influenced participant’s decision making and willingness to take risks [11]. To our knowledge, no studies have focused exclusively on understanding fear of depression recurrence (FoDR), defined in this study as having concerns, fears, or worries that one’s symptoms of depression will return or worsen at a future time.

Although an exploratory study, we aimed to investigate whether remitted depressed individuals experience FoDR, and if so, to explore the influence of these fears on daily functioning including coping, engagement in specific behaviours (e.g., avoidance, help-seeking), changes to cognitions and emotional states, and social patterns (e.g., withdrawal, seeking professional help). Exploring the concept of FoDR and its potential relationship to relapse, depression symptoms, and other indices of functioning may provide a better understanding of the unique cognitive and behavioural changes that occur following remission from an MDE and underlie depression recurrence. If found to influence important indicators of recurrence in MDD, FoDR may represent a novel phenomenon that can be targeted by future prevention and intervention efforts in MDD.

### The present study

This qualitative inquiry aimed to gain a better understanding of individuals’ experiences of FoDR. We used a social constructivist framework to guide this phenomenological study, given the important influence of culture, past experiences, social interaction, and context on an individuals’ beliefs about, and experiences with, MDD [26–28]. We conducted semi-structured interviews to: [1] identify whether remitted depressed individuals experience FoDR and evaluate the severity, frequency, triggers, and content of these fears; [2] explore how people respond to and cope with these fears; and [3] understand the impact of FoDR on daily functioning.

## Methods

This study was approved by the Human Research Ethics Committee at Concordia University in Montréal, Québec, Canada (REB# 30013399) and pre-registered on Open Science Framework (10.17605/OSF.IO/GQR2S). The pre-planned methodology was designed and reported in accordance with the Consolidated Criteria for Reporting Qualitative Research checklist (COREQ) [29] and the Standards for Reporting Qualitative Research (SRQR) [30].

### Participants and recruitment

English-speaking adults above the age of 18 years and in remission from MDD were recruited. First, participants who had previously completed a study in our lab were recruited via email. Second, we recruited participants from Québec institutions and mental health organizations using recruitment advertisements posted on social media platforms (e.g., Twitter, Facebook). As described in the Diagnostic and Statistical Manual of Mental Disorders (DSM, 5th Edition), participants were considered remitted from MDD if they reported a history of MDD and had been symptom-free for a consecutive period of at least two months [31]. Exclusion criteria included having [1] a major chronic medical illness highly associated with one’s past MDE; [2] a current and/or lifetime history of bipolar disorder I or II, a psychotic disorder (except if part of MDD), or a pervasive developmental disorder; and [3] a past or current comorbid Axis-1 disorder deemed to be one’s primary mental health diagnosis other than MDD.

### Materials and measures

*Mini International Neuropsychiatric Interview Version 7.0.2 (MINI)* [32, 33]. The MINI is a structured diagnostic interview used to assess DSM-5 mental disorders. It was used to assess for MDD, in remission, and to rule out the presence of any comorbid mental disorders. Psychometric evaluations of the MINI report satisfactory interrater reliability and concurrent validity with the Composite International Diagnostic Interview [34].

*Patient Health Questionnaire– 8 (PHQ-8)* [35]. The PHQ-8 is a validated self-report scale used as a diagnostic and severity measure for depression. Participants report how often they were bothered by symptoms of depression over the last two weeks, with higher total scores reflecting greater depressive symptoms. Participants reporting scores greater than 10 during our eligibility screening were excluded. The PHQ-9, which is psychometrically comparable to the PHQ-8, has excellent internal and test-retest reliability and adequate criterion and construct validity [36].

*Symptom Checklist 90– Revised (SCL-90-R)* [37, 38]. The SCL-90-R self-report scale evaluates psychological distress and symptoms of psychopathology across nine domains: Somatization, Obsessive-Compulsivity, Interpersonal Sensitivity, Depression, Anxiety, Hostility, Phobic Anxiety, Paranoid Ideation, and Psychoticism. The SCL-90-R also provides a Global Severity Index (GSI) which measures overall psychological distress. Participants indicated how much they were bothered by various symptoms over the past week. Higher scores reflect greater levels of pathological distress. The SCL-90-R has good internal reliability and high concurrent validity [39].

*World Health Organization Quality of Life Instrument-BREF (WHOQOL-BREF)* [40, 41]. The WHOQOL-BREF self-report scale measures quality of life (QoL) across four domains: physical health, psychological health, social relationships, and environment. Higher scores represent higher QoL. These domains have adequate internal consistency and test re-test reliability [41].

*Beck Depression Inventory (BDI-II)* [42]. The BDI-II is a 21-item self-report questionnaire evaluating current depression symptoms. Higher scores indicate greater depressive symptoms. The BDI-II has excellent internal consistency and test re-test reliability [43].

### Semi-structured interviews

Participants underwent a 60–90-minute semi-structured interview via *Zoom* [44] between October 2020 and January 2021. The first two authors of this paper (STG and AB), senior graduate students in clinical psychology, conducted and analyzed the interviews. The interviews were audio- and video-recorded and transcribed verbatim.

Our interview guide (see Additional File 1) was developed in our laboratory and inspired by existing FIR questionnaires and qualitative interview guides [45, 46]. The first section of the interview was comprised of open-ended questions about participants’ history of, and experiences with, MDD, FoDR, and the COVID-19 pandemic (data not reported). The FoDR questions explored the frequency, severity, content, triggers, and impact of participants’ fears and inquired about how participants respond to and cope with FoDR. Participants were also asked to generate a list of structured questionnaire items that will be used to develop a FoDR questionnaire. Our interview guide was reviewed iteratively by our research team until consensus on the items was reached.

### Procedure

Interested participants were invited via email to take part in an initial phone screening to obtain oral consent and confirm eligibility. If eligible, participants were provided with additional information about the study and invited to complete an online survey containing demographic questions and additional questionnaires on *SurveyMonkey* [47]. They then underwent the MINI and the qualitative interview. Recruitment ceased once data saturation had been achieved [48]. Participants were compensated $40.

### Data analysis

We used a content analysis approach to analyze the transcribed interview data, whereby repeated ideas and key concepts are labelled, coded, and categorized inductively from the data and integrated with existing literature [49, 50]. Content analysis enables the systematic and objective description and quantification of novel phenomena [51]. This approach allowed us to openly explore participants’ experiences with FoDR, while dually enriching our broader knowledge of FIR.

The first two authors (STG and AB) began by reading the transcriptions to fully immerse themselves in the data, before independently reviewing the first ten interviews word-by-word and assigning codes to every text fragment (i.e., units of meaning). The authors then reviewed these codes until consensus on labels was achieved and a preliminary coding scheme was developed. Then, both authors used this scheme to independently code the remaining twenty interviews. The first author (STG) then compared the two sets of codes obtained from coding the final twenty interviews and consensus was achieved through discussion with author AB until a final coding manual was established. Once all the interviews were coded, the investigators grouped codes capturing similar ideas into categories and subcategories. Quotations capturing thoughts that meaningfully expressed the core idea of each category were extracted and reported in-text and in Additional File 2. Coding was supported by the qualitative research software *ATLAS.ti* (Version 22.1.0) [52] and analysis of the demographic characteristics and psychosocial measures was conducted using the Statistical Package for Social Sciences (SPSS; Version 28) [53].

## Results

### Participant characteristics

Phone screenings were conducted with 51 participants, of which 36 were eligible to complete the questionnaires, MINI, and interview. Three participants did not complete subsequent parts of the study and three were excluded due to reporting current depressive symptoms. Thirty individuals (83% female; 37% White; Mean Age = 27.7) with remitted MDD completed the full study. Sociodemographic characteristics are presented in Table 1, mental health history in Table 2, and scores on the psychosocial measures in Table 3.

**Table 1:** Sociodemographic characteristics

Characteristics	Total (*N* = 30)
Female, *n (%)*	25 (83%)
Age in years, *mean (SD); range*	27.7 (8.96); 20–67
Race/Ethnicity, *n (%)*	
White	11 (37%)
East Asian	9 (30%)
Mixed	3 (10%)
Black/African American	2 (7%)
South Asian	2 (7%)
Middle Eastern	1 (3%)
Latino/Hispanic	1 (3%)
Southeast Asian	1 (3%)
Level of education, *n (%)*	
Postgraduate degree	5 (17%)
University degree	19 (63%)
Some college/CEGEP^a^	1 (3%)
Some university	5 (17%)
Occupational status, *n (%)*	
Employed	12 (40%)
Student	9 (30%)
Both a student and employed	5 (17%)
Unemployed	3 (10%)
Retired	1 (3%)

Note. ^a^Collège d’enseignement général et professionnel (CEGEP) is the post-secondary degree equivalent to grade 12 of high school and first year of university in the province of Quebec, Canada

**Table 2:** Participants’ mental health histories

Characteristics	Total *(N* = 30)
Number of depressive episodes, *mean (SD); range* (*n = 27*^a^; %)	7.33 (13.18); 1–60
One MDE	10 (37%)
Two MDEs	4 (15%)
Three or more MDEs	13 (48%)
Length of time since end of last MDE reported during qualitative interview, *range*	3 months– 14 years
Type of mental health professionals seen, n (%)	
Counsellor	15 (50%)
Psychologist	20 (67%)
Psychotherapist	9 (30%)
Psychiatrist	18 (60%)
Occupational Therapist	2 (7%)
Nurse	3 (10%)
Other (i.e., Ergotherapist, Community Worker, Medical Doctor)	3 (10%)
Level of disruption caused by worst depressive episode (*n = 27*^a^; %)	
Extremely disruptive	10 (37%)
Very disruptive	7 (26%)
Quite disruptive	9 (33%)
Somewhat disruptive	1 (4%)
Impact of current depressive symptoms (*n = 27*^a^; %)	
I am still experiencing symptoms	0 (0%)
I am experiencing much fewer symptoms than before	1 (4%)
I have no more symptoms OR I have some symptoms, but they do not bother me or interfere with my life	26 (96%)
Currently taking medication for depression, n (%)	
Yes	4 (13%)
No	26 (87%)
Received diagnosis other than depression, n (%)	
Yes	14 (47%)
No	16 (53%)
Self-Reported Comorbid Diagnoses, n (%)	
Anxiety Disorder (i.e., Generalized Anxiety Disorder, Social Anxiety)	8 (26%)
Eating Disorder (i.e., Anorexia, Binge Eating Disorder)	3 (10%)
Other (i.e., Obsessive Compulsive Disorder, Borderline Personality Traits)	2 (7%)

Note. ^a^Due to an experimenter error in setting up SurveyMonkey, three participants were not prompted to answer questions about the characteristics of their past MDEs

**Table 3:** Participant scores on the SCL-90-R, WHOQOL-BREF, and BDI-II

Scores on Measures of Psychosocial Functioning	Total (*N* = 30)
**SCL-90-R Scores**, *mean (SD); range (n* = 30)	
Global Severity Index Score	0.29 (0.26); 0–0.91
Interpersonal Sensitivity Subscale Score	0.38 (0.49); 0–1.56
Somatization Dimension Score	0.20 (0.19); 0–0.67
Obsession-Compulsion Dimension Score	0.48 (0.48); 0–1.50
Depression Dimension Score	0.45 (0.47); 0–1.62
Anxiety Dimension Score	0.17 (0.22); 0–0.90
Hostility Dimension Score	0.18 (0.24); 0–0.83
Phobic Anxiety Dimension Score	0.17 (0.30); 0–1.14
Paranoid Ideation Dimension Score	0.19 (0.38); 0–1.50
Psychoticism Dimension Score	0.14 (0.25); 0–1.00
**WHOQOL-BREF Scores**, *mean (SD); range (n* = 30)	
Physical Health QoL	16.0 (1.95); 11.43–20.0
Psychological Health QoL	14.6 (2.56); 7.33–19.33
Social Relationships QoL	14.58 (3.25); 6.67–18.67
Environment QoL	16.35 (1.82); 12.50–19.00
**BDI-II Scores**, *mean (SD); range (n* = 30)	5.73 (5.89); 0–22

Participants’ subjective reporting of the number of lifetime MDEs varied (*n* = 27; M = 7.33; SD = 13.18; Range = 1–60), with most experiencing two or more MDEs (*n* = 17; 63%). Almost all participants reported that they currently have no depression symptoms or that they have some symptoms that do not bother them or interfere with their life (*n* = 26; 96%). Due to an experimenter error, three participants were not prompted on *SurveyMonkey* to self-report specific MDD characteristics (see Table 2). Within the qualitative interviews, some participants (*n* = 24) indicated that their past MDEs ranged in duration (1 month– 20 years) and all participants reported being in remission for at least three months (3 months– 14 years).

### Qualitative findings

**Fig. 1:**
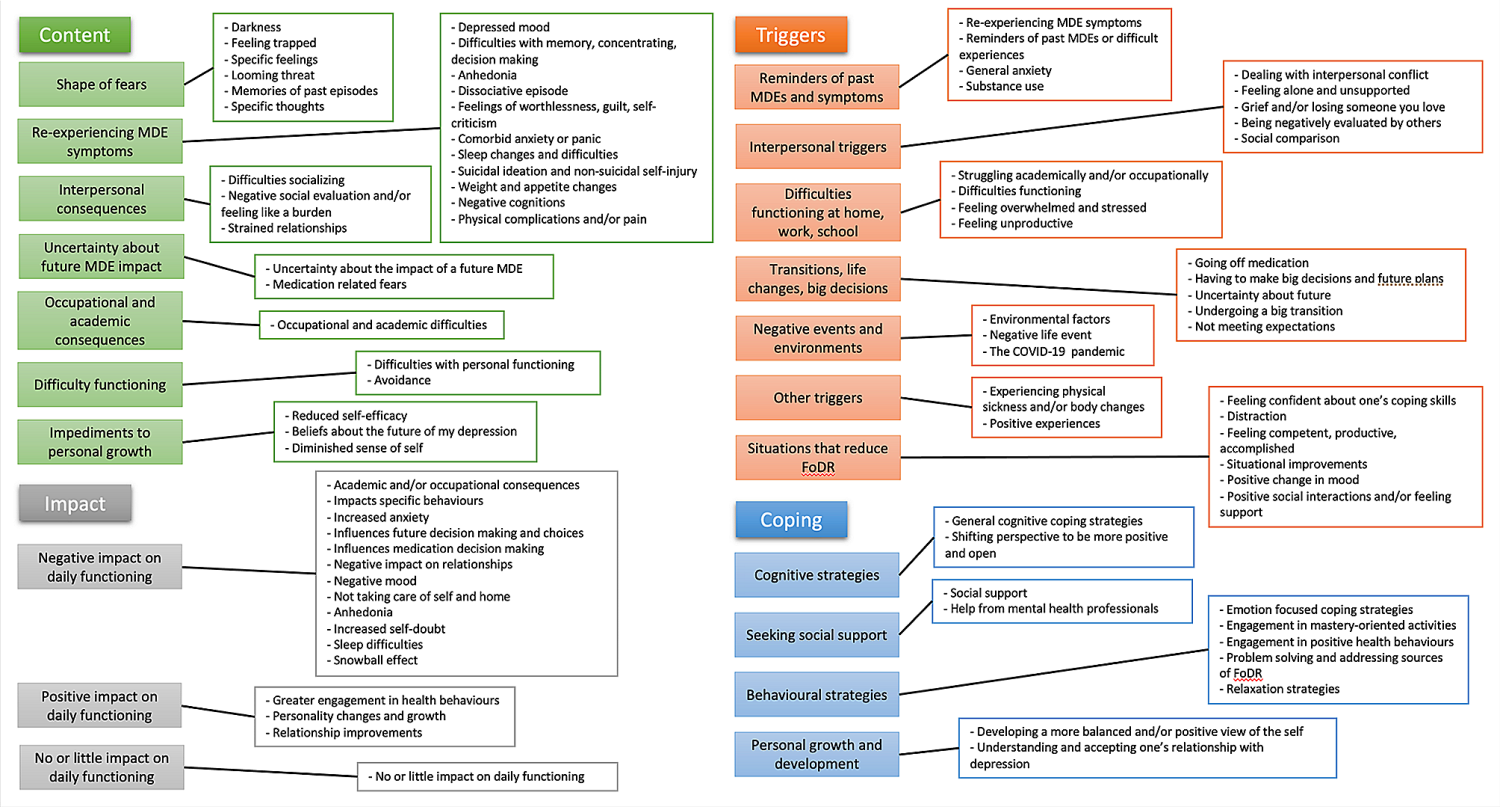
All FoDR categories, subcategories, and codes

We report here the most frequently mentioned codes. Reported “*n*” values refer to the number of times a code was mentioned by participants across the full interview. Reported percentages (%) refer to the percentage of our sample who reported the code at least once. Additional File 2 contains a complete list of all codes, categories, and subcategories and Fig. 1 contains a list of all FODR categories and subcategories.

#### Participants’ experiences with depression

*Triggers of past MDEs.* Participants reported interpersonal triggers of past MDEs, including social conflict (*n* = 32, 50%), feeling alone, isolated, and unsupported (*n* = 17, 47%), and relationship loss (e.g., grief, breakup; *n* = 11, 27%). Other MDE triggers included uncertainty about one’s future (*n* = 26, 47%), academic (*n* = 24, 53%) and occupational (*n* = 18, 30%) stressors, and transitions (*n* = 16, 30%).

*Symptoms and consequences of past MDEs.* All participants reported common MDD symptoms including depressed mood and negative cognitions (*n* = 61, 97%), sleep difficulties (*n* = 34, 80%), anhedonia (*n* = 36, 73%), feelings of worthlessness, guilt, and self-criticism (*n* = 26, 63%), weight and appetite changes (*n* = 19, 43%), and suicidal ideation and/or non-suicidal self-injury (*n* = 19, 33%). Reported consequences of past MDEs included difficulties functioning socially (*n* = 29, 63%), taking care of oneself (*n* = 15, 30%), and academic (*n* = 18, 37%) and occupational challenges (*n* = 7, 23%). Participant FD26 described the consequences of their MDE: “*I had to drop out of school, I had to stop working. I could barely take care of myself.”.*

*Coping strategies used to cope with past MDEs.* The most mentioned coping strategy to manage depressive symptoms was to seek help from a mental health professional (*n* = 37, 67%). Additional coping strategies included social support (*n* = 21, 40%), cognitive strategies (*n* = 16, 27%), medication (*n* = 15, 37%), and behavioural strategies (*n* = 16, 37%).

*Experience of being in remission from MDD.* When asked about remission, participants reported improvements to their mood (*n* = 22, 63%) and ability to take care of themselves (*n* = 8, 23%). For some, the experience of living through an MDE led to greater self-efficacy (*n* = 20, 50%), enhanced identity development and personal growth (*n* = 15, 33%), and a positive shift in one’s worldview (e.g., open-mindedness; *n* = 13, 30%). Some participants described experiencing distinct changes post-MDE including difficulty differentiating “normal” emotions (e.g., sadness) from clinical depression (*n* = 6, 20%) and continuous pressure to manage one’s mental health (*n* = 6, 13%).

#### Participants’ experiences with FoDR

*Presence, frequency, and severity of fears.* Twenty-two (73%) participants reported experiencing FoDR and eight (27%) indicated that they either do not experience FoDR or that their FoDR does not concern them. We explored differences in the presence of FoDR among the 27 participants in our sample who reported having either one past MDE (37%) or a history of two or more MDEs (63%). Among the 10 participants who reported having one past MDE, seven reported FoDR and three did not. For the 17 participants reporting a history of having two or more MDEs, 13 endorsed FoDR whereas four did not.

The frequency and severity of these fears varied. Some indicated that they have FoDR on a weekly (*n* = 12, 23%) or monthly (*n* = 10, 30%) basis, with these fears typically lasting a few minutes (*n* = 13, 43%) or hours (*n* = 7, 23%). Others reported that their fears remained persistent for days (*n* = 5, 17%). When asked to provide fear and distress ratings on a ten-point scale (1: “Not at all distressing/scared”, 10: “Extremely distressing/scared”), most participants reported distress ratings of five (*n* = 10) or six (*n* = 7), and fear ratings of three (*n* = 8).

*Content of fears.* Participants produced vivid descriptions to illustrate the shapes and sensations associated with their FoDR. For some, FoDR resembled specific memories of past MDEs (*n* = 11, 33%), an all-encompassing darkness (*n* = 9, 20%), or feelings of being trapped (*n* = 7, 17%). Participant FD22 described their FoDR as:

*“Terrible darkness. Like a hole where I’m going to fall into. And pain, a lot of pain. And if I fall into that all I will never be able to climb back. […]. It’s like a terror. It feels like I’m terrorized that if I fall, that’s it.”*

Participants expressed fears related to re-experiencing core MDE symptoms including depressed mood (*n* = 42, 70%), sleep difficulties (*n* = 25, 57%), anhedonia (*n* = 26, 40%), negative cognitions (*n* = 18, 37%), feelings of worthlessness, guilt, and self-criticism (*n* = 18, 40%), weight and/or appetites changes (*n* = 13, 30%), and suicidal ideation and/or non-suicidal self-injury (*n* = 12, 27%). Participant FD01 reported FoDR including:

*“Sleeping all the time and […] not maintaining my friendships and relationships. Not talking to anyone. […]. I won’t eat well, so then, like, I’ll gain weight, and then I just won’t like the way I look.”*

Participants’ FoDR were also centered around re-experiencing similar interpersonal consequences or challenges faced during past MDEs. These fears included experiencing difficulties socializing (e.g., feeling alone/isolated, withdrawing from others; *n* = 43, 63%) and negative social evaluation (e.g., burdening others; *n* = 32, 53%). Participant FD05 described:

“*For me it’s mostly feeling detached from everything. Because right now I feel like I’m in a situation where I’m surrounded by good people, my friends, my work environment, all of that. And I worry that if I go back to the depression, feeling emotionally detached from everyone, it’s just gonna ruin a lot of good friendships.”*

Participants expressed fears related to the uncertainty of how another MDE would impact their life (*n* = 37, 50%), including concerns about goal achievement, falling behind, and losing the progress made since their last MDE. Participant FD14 reported:

*“I would lose this happy life that I have right now. Like that just feeling okay would go away. So that’s definitely a scary thing.”*

Participants’ fears also included experiencing occupational and academic consequences (*n* = 30, 50%), difficulties with personal functioning (*n* = 24, 43%), and reduced self-efficacy (*n* = 14, 30%).

*Triggers of fears.* The two most frequently mentioned triggers of FoDR included re-experiencing MDE symptoms (*n* = 73, 83%) and reminders of past MDEs or difficult life experiences (*n* = 40, 60%). Some participants reported increased FoDR when struggling academically or occupationally (*n* = 33, 50%), when feeling overwhelmed and stressed (*n* = 23, 47%), and when experiencing difficulties functioning (*n* = 13, 23%). Examples of reported interpersonal triggers included dealing with interpersonal conflict (*n* = 27, 47%), feeling alone and unsupported (*n* = 20, 47%), and experiencing grief or loss (*n* = 9, 20%). Participants also described experiencing FoDR in response to uncertainty about the future (*n* = 23, 40%), having to make life decisions and future plans (*n* = 12, 17%), undergoing a transition (*n* = 10, 23%), and when facing negative life events (*n* = 20, 43%).

*Impact of FoDR on daily functioning.* Some participants reported a negative impact of FoDR on daily functioning. FoDR was associated with increased anxiety (*n* = 23, 57%), negative mood changes (e.g., sadness; *n* = 21, 47%), academic and/or occupational consequences (*n* = 16, 33%), and sleep difficulties (*n* = 5, 10%). Participant FD30 described that their FoDR:

*“Seem like a huge deal, I definitely can get a lot of anxiety over them. I can be really insanely upset over them. Again, like I’ve lost sleep over it.”*

Others described a “snowball effect” (*n* = 13, 37%), where FoDR led them to be increasingly hypervigilant to symptom changes, more overwhelmed, and at a greater perceived risk of MDE recurrence. FoDR also impacted participants’ engagement in specific behaviours (*n* = 15, 20%), including avoiding triggers of past MDEs, reducing responsibilities, and less risk-taking. For some, FoDR influenced future decision making and choices through exercising greater caution surrounding transitions and an increased sense of urgency when decision-making (*n* = 13, 17%). Participant FD08 describes how they:

*“Definitely don’t do the same things I used to before. Like, I’m not the same person, it’s been eight years. But I do feel like there was a shift in my personality from before and after my depression, like, I’m a lot less of a risk taker. I’m a lot less, you know, worry-free, I am more careful, I’m more aware.”*

FoDR also led to positive behavioural and personal changes. For some, FoDR led to greater engagement in health behaviours (n = 28, 40%), including trying to proactively address early warning signs of another MDE. FoDR was also associated with positive personality changes and growth (*n* = 17, 30%), including increased confidence and greater awareness of one’s mental health needs. Participant FD05 described how their FoDR:

*“Impacted [me] in a good way, because it makes me recognize some warning signs before they get bad. […]. If I’m maybe taking less care of myself, sleeping less, working too much until like I can’t focus on things anymore, I started to recognize that as a warning sign. And I kind of take a step back and focus on getting back to a structured routine, so it doesn’t get worse.”*

Some participants reported that FoDR had no or little impact on daily functioning (*n* = 28, 50%):

*“I don’t worry as much. The worry doesn’t last that long. So definitely no impact on my life. And emotionally too. […]. I wouldn’t say it affect[s] that much.”* [FD02].

*Coping with FoDR.* Participants commonly reported using cognitive strategies (e.g., acceptance, distraction; *n* = 79, 97%) and shifting their perspectives to be more positive and open (*n* = 23, 50%) to cope with FoDR. Participant FD20 described their coping style as:

*“Just more like accepting. Like, if it does come back […], I kind of like, know it’s not a forever thing. And I know, generally, like, I have strategies to help me.”*

Social support (*n* = 41, 67%), including interacting with friends, family, and engaging in social activities, was also helpful in managing FoDR. Participant FD12 described that their “*tool for addressing those thoughts coming back is just talking about it more.”.* Some participants used behavioural strategies to cope with FoDR including engaging in health behaviours (e.g., exercise; *n* = 35, 60%), directly addressing sources of FoDR (*n* = 14, 33%), relaxation strategies (e.g., mindfulness; *n* = 15, 37%), and participating in mastery-oriented activities (e.g., cleaning; *n* = 10, 23%). Others sought help from a mental health professional (e.g., therapy, psychiatrist; *n* = 15, 33%). Participant FD30 described their coping:

“*I write in a journal and I display my thoughts. So [my FoDR] doesn’t last more than a couple of hours I, if I’m really feeling scared or anxious, I will speak to someone, or I will check in with myself [as] I don’t want [it] to get to be something really big.”*

For some, understanding and accepting one’s relationship with depression (*n* = 37, 60%), including greater awareness of the signs of an incoming MDE, helped mitigate FoDR. Other FoDR coping strategies included developing a more balanced and/or positive view of the self (e.g., adjusting expectations; *n* = 27, 37%).

*Situations that reduce FoDR.* Participants reported that feeling competent, productive, and accomplished (*n* = 9, 27%), experiencing positive social interactions and/or feeling supported (*n* = 8, 27%), distraction (*n* = 8, 23%), and mood improvements (*n* = 8, 27%) reduced FoDR.

## Discussion

To our knowledge, this is the first study to explicitly examine FoDR among individuals with remitted MDD. Most of our sample (73%) reported FoDR, with these fears often occurring monthly or weekly and lasting minutes or hours. The most frequently mentioned triggers of FoDR included re-experiencing depression symptoms, reminders of past MDEs, and interpersonal conflict. The content of participants' FoDR varied, with participants most commonly reporting fears related to re-experiencing MDE symptoms, difficulties socializing, academic and occupational challenges, and uncertainty about the future.

There was substantial overlap between the reported triggers and content of participants’ FoDR. Participants described how negative interpersonal experiences, difficulties functioning at work, home, or at school, and re-experiencing MDE symptoms served as both triggers for and content of their FoDR. Other reported triggers included transitions, reminders of past MDEs, and decision making. These triggers overlapped with participants’ fears surrounding the uncertainty of having another MDE and the impact it would have on them. Although some content and triggers of FoDR were unique to depression, similarities emerged with what is known about FIR in cancer. For example, symptom changes (e.g., pain) and reminders of past cancer experiences are also documented triggers of FIR and some cancer survivors have endorsed fears related to the uncertainty of their futures, burdening others, and loss of independence [13, 54]. Similarly, participants in the present study described reminders of past MDEs as a key trigger of FoDR. Contrary to the fear of cancer recurrence literature, participants in this study did not report fears related to dying, experiencing physical pain, and undergoing treatments (e.g., chemotherapy) [54]. Notably, interpersonal factors, including conflict with friends and/or family and the potential loss of a relationship (e.g., grief, break ups), were reported as triggers of FoDR, representing factors that may distinguish FoDR from FIR more broadly.

Participants’ experiences of FoDR offer evidence for the cyclical nature of MDD. Both the triggers and content of participants’ FoDR were linked to the triggers, symptoms, and consequences of their past MDEs. Participants expressed concerns that if they were to have another MDE, they would have to endure the same symptoms and challenges previously experienced. Achieving full remission from depression is complex, and research investigating the course of depression is often criticized for a lack of consensus about key points of change within the depression cycle [55, 56]. Although at the time of the study all participants met criteria for remission from MDD, our findings indicate that the impact of having endured an MDE (e.g., life consequences, behavioural changes), influenced participants’ FoDR. These findings support the idea that deficits in functioning and other longstanding consequences of MDEs (i.e., changes in self-perception) may remit slower than acute MDE symptoms and contribute to the resurgence of residual symptoms and MDE recurrence through means of FoDR [57, 58]. Indeed, the presence of residual symptoms is a well-established and robust predictor of MDE recurrence [10], providing support that the present fears around past depressive symptoms and consequences may signify risk for relapse.

Participants used similar coping strategies in response to FoDR that they reported using during past MDEs (e.g., cognitive strategies, professional help). Participants indicated that reflecting on their experiences with MDD helped them to develop a more balanced and positive view of themselves and accept their depression histories, which, in turn, helped them cope with FoDR. Similar coping strategies have been reported in other qualitative studies of depression [59–61]. However, it was sometimes challenging for participants to discern when in the depression cycle specific coping strategies were used (i.e., during past MDEs, in response to FoDR, in the ongoing management of one’s general mental health, or to cope with recurrent symptoms). These findings suggest that we have yet to understand how and when in the depression cycle these strategies are used.

The impact of FoDR on participants’ daily lives varied substantially. Notably, 50% of our sample reported that FoDR had little or no impact on their lives. For others, FoDR had a negative impact, including posing challenges to decision making, negatively affecting one’s mood, academic and occupational consequences, and functional impairments. Conversely, some participants reported that FoDR positively impacted their lives by fostering personal growth and motivating them to engage in health behaviours to reduce the risk of MDE recurrence. Similar impacts of FoDR on functioning and self-perceptions were described by Coyne et al. [11] who found that living through an MDE provided some participants with a renewed sense of strength, whereas others felt pressured to continuously manage their mental health and reduce risk taking. The multidimensional impact of FoDR reported in this study complements what we know about FIR in cancer. Fear of cancer recurrence has been associated with greater depression and anxiety symptoms, reduced quality of life, reduced engagement in social activities, and limited coping [13, 17, 62, 63]. Positive influences of FIR in cancer have also been reported including serving as a motivator to better manage one’s illness through self-care, positive coping, and symptom monitoring [13, 19].

### Future directions

Identifying the factors that influence how one responds to FoDR will help us differentiate why some individuals report no impact or a positive impact of FoDR whereas others describe a negative influence on daily functioning. Future research should explore whether individual (e.g., neuroticism, FoDR severity, coping) and/or characteristics of the disorder (e.g., duration and severity of past MDEs) influence the presence and impact of FoDR on daily functioning, mood, cognitions, and behaviours. For example, 26% of our sample reported having a comorbid anxiety disorder and 48% endorsed a history of more than three past MDEs. It would be beneficial to examine whether a history of recurrent MDD and/or having an anxiety disorder are contributing to the presence of FoDR and its negative impact on functioning. Further, identifying the factors that explain the variance in how our participants responded to and perceived their FoDR may allow for the development of more tailored treatment and relapse prevention protocols for individuals with remitted MDD endorsing FoDR. This knowledge may also provide an avenue to further explore potential “at risk” profiles of individuals with remitted MDD who may be at higher risk of MDE recurrence through engagement in specific cognitive and behavioural changes associated with FoDR. Similarly, knowledge of the underlying characteristics leading individuals to respond positively to FoDR (e.g., personal growth, greater engagement in health behaviours), may lead to the identification of targetable factors in relapse prevention interventions for recurrent MDD.

The substantial overlap between the content and triggers of participants’ FoDR with their past MDE experiences, confounded by the presence of residual symptoms, offers evidence for the cyclical, dynamic, and constantly changing nature of MDD. Given this, we recommend viewing FoDR as a dynamic construct that may wax and wane in intensity and severity as one progresses through the depression cycle. Thus, it is important to examine the presence and impact of FoDR longitudinally, to explore whether FoDR and one’s response to these fears may evolve based on disorder-specific, environmental, situational, and individual changes.

Future research should also consider the similarities and differences of the nature and impact of FIR across both psychiatric (e.g., depression, psychotic disorders) and medical conditions (e.g., cancer). For example, perhaps people’s perceptions of their illness and the level of perceived control they have over the resurgence of symptoms may differ dramatically between those with a psychiatric condition (i.e., depression) versus a physical one (i.e., skin cancer). These potential differences may then inform the presence of FoDR and the extent of impact associated with these fears. Finally, future FIR research would benefit from interdisciplinary research collaborations aimed at identifying the elements of FIR that may present transdiagnostically across both psychiatric and medical conditions.

Given the intersecting influence of an individual’s past MDE on FoDR, future research should focus on disentangling how residual depression symptoms and consequences of past MDEs relate to FoDR and influence MDD prognosis. To do this, it may be beneficial to include participants endorsing residual symptoms of MDD within future studies of FoDR. However, prior to being able to quantitatively examine FoDR and its relationship to health outcomes, personality characteristics, and other indices of functioning, a psychometrically valid measure of FoDR must be developed. This measure should be designed to capture the unique elements of MDD that are not otherwise represented across measures of FIR in cancer and other chronic illnesses.

### Limitations

Several limitations are worth noting. Firstly, all interviews were conducted online, which may have influenced the willingness of participants to speak candidly about their experiences. Secondly, although our sample was adequate in size and we achieved data saturation, we did not conduct subgroup analyses with unique depression subtypes (i.e., recurrent vs. single episode MDD) or based on any sociocultural factors. Therefore, we are unable to draw conclusions about whether there are differences in the experiences of FoDR across clinical and sociocultural variables. Thirdly, some participants, particularly those with long durations since the end of their last MDEs, may have experienced memory bias, thus influencing the accuracy of their ability to recall important details about their past MDEs and current FoDR. However, we chose to recruit broadly to ensure that our final sample contained participants with a wide range of experiences and diverse characteristics. Fourthly, we did not use a quantitative estimate of intercoder reliability, which may limit the objectivity and reliability of our analyses and interpretations. However, aligned with the recommendations proposed in the COREQ [29] and SRQR [30] guidelines to enhance credibility, our data analysis approach involved multiple coders, clear descriptions of how we engaged with and analyzed the data, and transparency in how we developed our final codebook. We also included many supporting quotations from different participants both within our results and in Additional File 2 to enhance the transparency and trustworthiness of our findings and interpretations of the data.

Finally, although we used both the MINI diagnostic interview and the PHQ-8 to screen out participants with current depressive symptoms, results from the self-reported BDI-II scores indicated that some participants endorsed minimal depressive symptoms during the study. It is possible that having current depressive symptoms confounded a participants’ experiences with FoDR. It is also possible that not including participants with residual symptoms compromised the generalizability of our findings as it is common for individuals in remission from MDD to endorse the presence of and fluctuations in residual symptoms. These findings also highlight the importance of differentiating FoDR from residual depression symptoms.

## Conclusions

We used semi-structured interviews to gain a thorough and nuanced understanding of the different content, triggers, severity, and impact of a participants’ FoDR. Our findings paralleled what we know about FIR in other health conditions and uniquely captured the lived experience of individuals with remitted MDD. Understanding the diverse impact that FoDR has on daily functioning and MDD prognosis may present a window into understanding the mechanisms influencing MDE recurrence.

### Electronic supplementary material

Below is the link to the electronic supplementary material.

Additional file 1: Qualitative interview guide

Additional file 2: Codebook containing all codes, categories, subcategories, code definitions, example quotations, and number of code mentions

## Data Availability

The datasets generated and analyzed during the current study are not publicly available due to concerns about revealing the individual privacy and identities of participants but are available from the corresponding author on reasonable request.
