# Supplementary material for: Fear of depression recurrence among individuals with remitted depression: a qualitative interview study
Source: BMC Psychiatry. 2024 Feb 21;24:152. doi: 10.1186/s12888-024-05588-4 (PMC10882790; doi:10.1186/s12888-024-05588-4)
Supplement: Supplementary file 1 — Additional file 1: Qualitative interview guide [file 12888_2024_5588_MOESM1_ESM.docx]

**Additional File 1. Qualitative Interview Guide**

**Begin the Interview:**

We are interviewing you today because we are interested in knowing more about your experiences with no longer being depressed and about whether you ever experience thoughts about becoming depressed again. We are interested in hearing more about your stories with depression, as well as any thoughts and feelings you may have about your depression returning.

Sometimes people who undergo interviews like these are unsure of what is of interest to researchers, but please feel free to speak about anything that you think is relevant to the question and important for you. After all, the more information we can gain from you about this topic, the more informative it will be for us!

**Interview Questions**

**Part 1 – History of and Experiences with Past Depressive Episodes:**

These first few questions will inquire about your past experiences with depression. To begin:

1. Can you tell me a little bit about your last depressive episode?
   1. How long ago was it?
   2. How long did it last?
   3. How did you feel at that time?
2. How many depressive episodes would you say you’ve had in the past?
   1. *If more than one*: Of all of your past depressive episodes, did one stand out as being worse than the others?
      1. What do you think contributed to making this depressive episode worse than other one’s you’ve had?
3. How long have you felt free from most symptoms of depression?
4. What has it been like for you to no longer feel depressed?

**Part 2 – Assessing Fear of Recurrence:**

These next questions will ask you about whether you ever think about becoming depressed again. Specifically:

1. Do you ever worry that your depression might come back?
2. **If no**, what do you think helps you from not worrying about whether your depression might return?
   - 1. *Prompts (if needed):* Are there certain things you tell yourself or techniques or strategies that you find helpful?
        - For EACH coping strategy ask participants to rate the helpfulness of that strategy on a scale from 1 to 10 with 1 being extremely ineffective and 10 being extremely effective.
          1. List the strategies and ratings here:
          2. Strategy: ___________________________
          3. Rating: _________
          4. Strategy: ___________________________
          5. Rating: _________
          6. Strategy: ___________________________
          7. Rating: _________
     2. If you were to give advice to someone who worries that their depression will come back, what would you tell them?
        - *Prompts (if needed):* What strategies would you share with others who worry that their depression might come back?
3. **If yes**, what do these thoughts or images about your depression coming back typically look like or sound like to you?
   - 1. **Frequency:** How often do you have thoughts or images about your depression returning?
        - *Prompt:* Daily? Weekly? Once a month?
     2. **Length:** Once you start to worry about your depression returning, how long does this thought or image typically last?
        - *Prompt:* A few minutes? A couple of hours? All day? All week?
     3. **Distress:** On a scale of 1-10 how distressing would you say this thought is to you with 1 being not at all distressing and 10 being extremely distressing?
     4. **Nature of Worries:** Are there specific aspects of your depression that you worry may return?
        - *Prompt (if needed):* For example, are there specific symptoms or past consequences of your depression that you worry may return?
        - *Prompt further (if needed):* For example, perhaps you are worried that if you get depressed again you will have a hard time getting out of bed in the mornings, or maybe you worry that you will have to take another leave of absence from work?
        - ***Prompt (interpersonal worries, if needed):*** For example, perhaps you are worried that if you get depressed again you will disappoint those you care about, such as a partner, friends, and family?
          1. *If parent (prompt):* Perhaps you are worried that if you get depressed again you will be unable to care for your children?
          2. *If in a relationship (prompt):* Perhaps you are worried that if you get depressed again, this will affect your relationship with your partner and/or you might feel like a burden to them?
     5. **Triggers (Situations):** Are there any specific situations or contexts that increase the worries you have about your depression returning?
        - *Prompt (if needed):* For example, if you are in a certain place or around certain people do you notice that these thoughts get worse?
        - **Then ask:** Are there any situations that lead to you feeling less worried about your depression returning?
     6. **Triggers (Feelings/Thoughts):** Are there any feelings that increase the worries you have about your depression returning?
        - *Prompt (if needed):* For example, if you are feeling a certain way, such as sad, or even happy, do you notice that these thoughts get worse?
     7. **Impact of Worries:** Do you think that worrying about your depression returning has impacted your life in any way?
        - **If yes**, in what way?
          1. *Prompts (if needed)*: For example, do you notice that these worries impact your mood or behaviour, or even your desire to engage in certain activities?
          2. *Prompt further:* Ask about impact on emotions, thoughts, behaviours, and functioning in everyday activities, work, social contexts, etc.
     8. **Response to Worries:** When you notice yourself beginning to worry that your depression may return, how do you typically react to these worries?
        - *Prompt (if needed)*: For example, what happens in the moment when you start to worry that your depression might return? Can you walk me through it?
        - *Prompt further (if needed)*: When you notice yourself worrying that your depression may return, how does it make you feel? How do you react to these worries? Is there something you do?
     9. **Coping:** Are there things that you do to try and manage the worries you have about your depression returning?
        - **If yes,** what are they?
          1. *Prompt:* For example, some people may try to distract themselves from uncomfortable thoughts or they may seek support from friends and/or family members.
          2. For EACH coping strategy ask participants to rate the helpfulness of that strategy on a scale from 1 to 10 with 1 being extremely ineffective and 10 being extremely effective.
          3. List the strategies and ratings here:
          4. Strategy: ___________________________
          5. Rating: _________
          6. Strategy: ___________________________
          7. Rating: _________
          8. Strategy: ___________________________
          9. Rating: _________

**For All Participants:**

1. **Participant Rating:** On a scale of 1-10 with one being not at all scared and ten being extremely scared – how scared would you say you are about your depression coming back?
   1. **Participant Rating (1-10):** _______
2. **Interviewer Rating (silent):** Please provide a subjective rating of the severity of the patient’s fear of depression recurrence on a scale from 1-10 with the following response options:
   1. **0** - Don’t think about it, no avoidance, no disturbance associated
   2. **1-2** - Don’t think about it, efforts to dismiss, no disturbance associated
   3. **3-4** - Rarely think about it, efforts to dismiss, low emotional disturbance, no functional impairment
   4. **5-6** - Sometimes think about it, efforts to dismiss, moderate emotional disturbance, low functional impairment
   5. **7-8** - Think about it a great deal, efforts to dismiss, high emotional disturbance, moderate functional impairment
   6. **9-10** - Think about it constantly, efforts to dismiss, high emotional disturbance, high functional impairment

**Part 3 – COVID-19 Specific Question:**

1. Finally, do you think that the current COVID-19 pandemic and the impact it may have had on your daily living has influenced the worries you may have about your depression returning?
   1. If **yes**, how do you feel the COVID-19 pandemic has influenced your worries about your depression returning?
   2. If **no**, is there anything in particular that you have done during the COVID-19 pandemic that would influence you not worrying about your depression returning?

**Part 4 – Structured Item/Idea Generation**

Thank you so much for answering these questions. Our next and last section will be a bit more structured as we will ask you to come up with a list of potential ideas for a questionnaire that we are developing. More specifically, one of the secondary goals of this study is to use the information you have provided to us today to develop a questionnaire that can assess the extent of worry someone has about their depression returning and the specific triggers and consequences of those worries. This questionnaire will be used to help identify and address any worries about depression recurrence that people may have prior to them ending treatment, such as therapy, so that mental health practitioners can better address these concerns and fears early on in the therapy process.

**To begin:**

1. We just spoke about some of the different feelings or situations that may provoke feelings of worry about whether your depression will return. If you had to make a list of the top five potential “triggers” that may lead to you worrying about your depression returning, what would they be? *Prompt (if necessary):* remember, these can be specific thoughts, feelings, situations, or even reminders of past symptoms and consequences.
   1. 1) ___________
   2. 2) ___________
   3. 3) ___________
   4. 4) ___________
   5. 5) ___________
2. Similarly, if you had to make a list of the five most relevant aspects of your depression, including symptoms or past consequences, that you worry may return or happen again, what would they be?
   1. 1) ___________
   2. 2) ___________
   3. 3) ___________
   4. 4) ___________
   5. 5) ___________
3. Finally, if you had to make a list of the five biggest impacts that worrying about your depression returning has had on your current life, what would they be?
   1. 1) ___________
   2. 2) ___________
   3. 3) ___________
   4. 4) ___________
   5. 5) ___________

**Part 5 – Wrap Up**

That concludes the interview for today. Before we wrap up, is there anything else that you think would be important for us to know about you and your experiences with depression?

Thank you very much for taking the time to answer our questions today, we really appreciate it and have learned a lot from you. Before we hang up, do you have any final questions about the interview today or about the study in general?
