# Supplementary material for: Fear of depression recurrence among individuals with remitted depression: a qualitative interview study
Source: BMC Psychiatry. 2024 Feb 21;24:152. doi: 10.1186/s12888-024-05588-4 (PMC10882790; doi:10.1186/s12888-024-05588-4)
Supplement: Supplementary file 2 — Additional file 2: Codebook containing all codes, categories, subcategories, code definitions, example quotations, and number of code mentions [file 12888_2024_5588_MOESM2_ESM.docx]

**Additional File 2.** **Codebook containing all codes, categories, subcategories, code definitions, example quotations, and total code mentions.**

| **Category, Subcategory, & Codes** | **Code Definition** | **Example Quotation** | **Total # of Mentions** | **N (%) of Sample** |
| --- | --- | --- | --- | --- |
| **Section 1: Participants’ Experiences with Depression** | | | | |
| **Category: Triggers of past Major Depressive Episodes (MDEs)** | | | | |
| **Subcategory: Interpersonal or social triggers** | | | | |
| Feeling alone, isolated, and unsupported | Any mention of participants’ past MDEs being triggered by feelings of loneliness, isolation, and/or lack of support. | *“Again, like time away from family, I do live four hours away. So it felt a lot like I couldn't reach out to my parents, even if I try. University just seems a lot tougher than high school, like my grades have been a bit worse in high school. So that puts a lot of strain on me. And I put a lot of pressure on myself and I'm a lot harder on myself than I was in high school. Other factors can be just like I love my friends here, but my friends at home know me and they know my struggles a lot better obviously, because I've been friends with them for a lot longer so they can help me a lot more. And they don't have any friends from home here. So I just, I think a lot of isolation. And then once I'm isolated, I kind of like just dig the hole deeper. So it was a quite intense time.”* [FD30] | 17 | 14 (47%) |
| Relationship loss | Any mention of participants’ past MDEs being triggered by a loss of a relationship (e.g., grief, breakup, rejection). | *“But... Yeah. It was after hard breakup. So it was definitely triggered, triggered by by an event. And yeah, I mean, it's mostly because I was I was struggling to I was struggling with a breakup.”* [FD03] | 11 | 8 (27%) |
| Social conflict | Any mention of participants’ past MDEs being triggered by ongoing interpersonal conflict with friends or family. | *“I cannot concentrate in when I go back home, it was even worse because I have to face like my parents arguing. And they're arguing very aggressively. Like my dad was violent. He was swearing. And my mom would cry. And then like, it's not it was not about me, it was about them, their issues. But because I was young, I felt like I was part of it like because they're my parents. But now I grow up I understand their issues. It was not... it was really purely their issue, like their personality, their family and everything was conflicting, so... But when I was young, I don't understand any of that. So it was very hard. And then also my dad like... cheated. And also like broke my mom a lot. And I was really close to my mom growing up. I spent most of time with my mom but not my dad because he's always working. So that was very, very shocking, like a big shock to me.”* [FD02] | 32 | 15 (50%) |
| **Subcategory: Occupational, academic, or goal related triggers** | | | |  |
| Academic stressors | Any mention of participants’ past MDEs being triggered by school stressors (e.g., difficulties managing one’s workload, burnout). | *“So I was having difficulties in school for like, the first time in my life, during University. And I wasn't really sure kind of what to do to improve or kind of what my next steps would be. I was feeling pretty hopeless.”* [FD17] | 24 | 16 (53%) |
| Occupational stressors | Any mention of participants’ past MDEs being triggered by occupational stressors (e.g., overwhelmed by workload, loss of job, job rejections). | *“So I love reading and it was a job at [XXX]. And I was really excited about it. And I was actually like, hired into kind of like a mini manager role. So I was really proud of that. And then they just kind of said, You know what, this isn't working. Please don't come back. And I just felt stuck. Like that was the word that I used. I felt stuck in this life. Nothing was going the way I wanted it. Nothing was happy. Like friend, boyfriend, school, work. Everything was miserable. I was lonely. And I just felt very stuck.”* [FD16] | 18 | 9 (30%) |
| Financial stress | Any mention of participants’ past MDEs being triggered by financial stress. | *“And I think I just, especially because I was also the one paying for myself, it was even more expensive than my university fees now. So I was like, I'm not doing well. I kind of like always identified as being the smart kid.”* [FD20] | 4 | 4 (13%) |
| Uncertainty about one’s future | Any mention of participants’ past MDEs being triggered by experiencing uncertainty about one’s future goals and ability to achieve them (e.g., feeling insecure, directionless, trapped/stuck, worrying about the future). | *“I think at that time, I was just lost, I felt like everyone knew what they were doing, why they weren't going and I was just frozen in place. And nothing mattered because no matter what I did, I wasn't going to be happy. I wasn't going to find something that I could be happy doing.”* [FD05] | 26 | 14 (47%) |
| Transitions | Any mention of participants’ past MDEs being triggered by undergoing a big transition. | *“I think I was 12, I think I think I was 12 years old, because that was the time I moved on from elementary school to middle school because Korea has elementary, middle school and high so that it was a lot of change. There were there were a lot of changes. And that was also time when my family was most broken possible. It was very hard. There were... it was a lot of things happened over over the whole, like, it was really long, a lot of things happen over that period of time.”* [FD02] | 16 | 9 (30%) |
| **Subcategory: Situation specific triggers** | | | |  |
| Bad living conditions | Any mention of participants’ past MDEs being triggered by difficult living conditions. | *“Yeah. Yeah. And there was another episode that was caused by one of my I was living in a small condo at that time. And I had a terrible neighbor that was always telling me stupidity and being very unpleasant. Almost harassment.”* [FD22] | 2 | 2 (7%) |
| COVID-19 pandemic stress | Any mention of participants’ past MDEs being triggered by the COVID-19 pandemic and any associated stress. | *“So first of all, I would say like the the COVID situation, as unique as it is, but maybe like, just something that, like you guys, as you're doing the research at this point in time in history, I think, at least for me, it's been the biggest part, or the biggest factor. And like, it just took away, like the social interactions, like the day to day interaction with friends, or like opportunities for you to engage in like physical activities or things like that. So all that like things that used to bring me joy are no longer, like, readily available. So to me, that is a big part of it.”* [FD34] | 5 | 5 (17%) |
| Postpartum | Any mention of participants’ past MDEs being triggered by postpartum experiences. | *“So that was the main trigger along with. I wasn't sleeping very well, for the first like, three years, no, six months before we went to the Philippines. And I think like my choices were wrong. My priorities were wrong. I basically didn't have a backbone.”* [FD11] | 2 | 1 (3%) |
| Puberty | Any mention of participants’ past MDEs being triggered by undergoing puberty and its related effects. | *“Um, I was I was very young. I mean, I was, I was in high school, I was probably 13-14. 14 maybe. But it was, but it was. I mean, I look back on it as a depressive period. But, you know, I, there was also all these teenage emotions and anxiety. And I don't know, it's hard. It's hard to say now that I'm an adult, it like, but to me, it's, it was a depressive time. I just didn't I mean, I was growing up and didn't know how to like, I was just questioning everything, questioning life, and I didn't know how to deal with my emotions and and I just felt worthless. Yeah, they were just like, really, I had really, really bad thoughts about myself.”* [FD30] | 3 | 3 (10%) |
| Seasonal | Any mention of participants’ past MDEs being triggered by seasonal changes. | *“Um not necessarily, I think the only thing that I can really like, say had an impact is I'm like, affected by like the seasons. So winters are always a lot harder. But it wouldn't necessarily be like, oh, when it's winter, I'm depressed. When it's summer, I'm feeling better. It was just kind of in summer, I typically feel a lot better. And in the winter, I typically feel worse, but like those like, like downward peaks can kind of be anywhere. It's just like, on average, that's the best way that I can describe it.”* [FD26] | 2 | 2 (7%) |
| Medication changes | Any mention of participants’ past MDEs being triggered by medication changes. | *“*When *I first started medication, I had pretty adverse side effects to the first thing that I was put on. It was not great. I had like kind of a big flip flop of going from like a ton of fatigue, low appetite to just like come the complete opposite, like eating a lot, being awake all the time, being really like hyper aware, and I didn't like it at all. And so there was like this thing of like, I've never even I never have been on like any sort of medication long-term other than birth control. And I didn't really have any side effects when I started birth control. So I was like, dealing with my body feeling different.”* [FD36] | 2 | 2 (7%) |
| Loss of routine resulting in too much time to think | Any mention of participants’ past MDEs being triggered by a loss of structure and having too much time to think. | *“But then I got to having this feeling that I completely wasted my first year. So I came back home, and I kind of wish I wasn't home. And I had nothing to do really, I was just like, having a... And and I just started feeling sad all the time. But I would call it I would just, I wouldn't want to do anything. I would just sit at home. I couldn't even watch TV nothing or just like, be there.”* [FD07] | 5 | 2 (7%) |
| **Subcategory: Other triggers** | | | |  |
| Lack of understanding of depression and how to cope with it | Any mention of participants’ past MDEs being triggered by not understanding depression and knowing how to cope with it. | *“I had no way to cope with it. I I didn't know that there were things that I could do to make, to make it better, people I could talk to, know what I could do and the resources I had, it was just, I thought that there was something wrong with me that I was always going to be like that. I didn't know who to talk to, where to go. And everywhere I thought of to cope with it was making it worse.”* [FD05] | 6 | 6 (20%) |
| Comorbid mental health symptoms | Any mention of participants’ past MDEs being triggered by comorbid mental health symptoms (e.g., trauma, substance use, anxiety, eating disorder). | *“I think for me, my my depression was definitely a result of, or at least flared up by the sexual assault that I mentioned.”* [FD33] | 16 | 10 (33%) |
| Unstable sense of self | Any mention of participants’ past MDEs being triggered by having an unstable sense of self. | *“Also, I think now I look back, I think also I was going through like puberty. So I was really trying to find for myself, like who I am and everything, but it was not. I remember, when I went to try to find myself, I find myself very not fit to the normal people in the society. So I was... it was very hard for me to accept. So I was think instead of accepting myself, I would just going around and round and just keep denying No, no, no. But by doing that, it was also... it doesn't feel good.”* [FD02] | 6 | 6 (20%) |
| No trigger | Any mention of participants’ past MDEs not having an identifiable trigger. | *“All of a sudden, it really just happened out of the blue, like nothing happened in my life to trigger it.”* [FD12] | 2 | 2 (7%) |
| **Category: Symptoms and consequences of past MDEs** | | | |  |
| **Subcategory: Symptoms experienced during last MDEs** | | | |  |
| Depressed mood and negative cognitions | Any mention of experiencing depressed mood (e.g., feeling low, down, angry, sad, hopeless) and/or negative cognitions (e.g., negative outlook, rumination) during a past MDE. | *“It was a bit mixed like I was, yeah. Because the anger was having more effect on my personal life like social life. Sadness is because like, I don't really show it to other people. I keep it to myself. So I guess people mostly saw like me being irritable. And but yeah, I was like, really sad and crying a lot.”* [FD28] | 61 | 29 (97%) |
| Anhedonia (i.e., diminished interest or pleasure in activities) | Any mention of experiencing diminished interest or pleasure in activities (i.e., anhedonia) and low motivation during a past MDE. | *“And then I would talk to someone and I would realize how how like I had the stuff I needed to do and things that I should be doing. And then I'd be angry at myself for not being able to do them on time. And then I'm like why are you like this... And it's just, it's like it would be like alternate between being angry at myself and then not being able to do anything. And just be like, urgh.”* [FD01] | 36 | 22 (73%) |
| Feelings of worthlessness, guilt, and self-criticism | Any mention of feeling worthless, guilty, or engaging in self-criticism during a past MDE. | *“Um like anything, and at some point after being depressed for years, I kind of started to think, you know, maybe I'm not depressed, maybe it's just me, it's who I am. It's how I am. Maybe I'm just, maybe I'm failing out of my classes, because I'm just not smart. Maybe I'm never gonna be able to handle the job or handle everyday life, like everyone seems to. Maybe it's just something wrong with me.”* [FD05] | 26 | 19 (63%) |
| Sleep difficulties | Any mention of experiencing sleep changes or difficulties, including fatigue, during a past MDE. | *“Yeah, definitely overslept. I was feeling more tired than usual. And also as a way to escape everything, escape my thoughts. I took a lot of naps, and I wanted to sleep all the time.”* [FD12] | 34 | 24 (80%) |
| Suicidal ideation and non-suicidal self-injury | Any mention of experiencing suicidal ideation, suicide attempts, or engaging in non-suicidal self-injury (NSSI) during a past MDE. | *“You know, I had sort of very classic symptoms, you know, I slept way too much, you know, and I ate too much, or I didn't need enough. You know, I felt sad, and it was sad. All the time. I thought about death. I thought about killing myself. I was self-injuring.”* [FD14] | 19 | 10 (33%) |
| Weight and appetite changes | Any mention of experiencing weight fluctuations and/or appetite changes during a past MDE. | *“And, um, during that time, I stopped eating. And every time I ate, the food would come back up. And I, I spent most of my days just sleeping. And so while I'm sleeping, I don't eat. So then during that period, I lost like 30 pounds, around 30 pounds, within one month. And I lost, like, a significant amount of weight. And it showed. I was neglecting work because of it. Like I sometimes I would just call in and say like, I'm not showing up, and stay home sleep. Yeah, I will I I wasn't sleeping. I wasn't eating. And every time I tried to eat I, it wouldn't stay down, like my body would reject food. Yeah. um not sure what else to say about it.”* [FD32] | 19 | 13 (43%) |
| Comorbid anxiety | Any mention of experiencing comorbid anxiety or panic during a past MDE. | *“But it was mostly anxiety, I couldn't sleep, I couldn't eat. I was nauseous all the time. And because of the anxiety, I started feeling very down and depressed. But it didn't last very long, because I kind of recognized it. So I, you know, went to see my therapist, and you know, I was able to deal with it.”* [FD08] | 6 | 5 (17%) |
| Difficulties thinking, concentrating, or making decisions | Any mention of experiencing difficulties concentrating or making decisions during a past MDE. | *“But I was still coming out of like, a really big, like burnout. So I still had a lot of the side effects that I wouldn't necessarily say were caused by depression, but like that I had during depression, so trouble concentrating, having to sleep a lot more. Just kind of stuff that I need to work on retraining myself to be used to.”* [FD26] | 7 | 7 (23%) |
| Hospitalization | Any mention of being hospitalized during a past MDE. | *“And depression for me, as I said, lasted about 20 years. In that time, you know, almost every day, I was sad, I cried multiple times. I was hospitalized on four different occasions, I had four suicide attempts that didn't necessarily correspond with the hospitalizations but, you know, around the same time, I was followed by a psychiatrist for many, many years.”* [FD14] | 3 | 3 (10%) |
| Psychomotor agitation or retardation | Any mention of experiencing psychomotor agitation (e.g., fidgeting) or retardation (e.g., inability to speak) during a past MDE. | *“Yeah. So then I kind of messaged my boyfriend, and I said, you need to take me to the hospital. So he came, and he got me and he brought me to the hospital. And I at this point was, like, nonverbal. Like, I wasn't talking. And he would ask me a question and it was too much effort to answer. So I just didn't answer.”* [FD16] | 2 | 2 (7%) |
| Somatic symptoms | Any mention of experiencing somatic symptoms (e.g., nausea, vomiting) during a past MDE. | *“And yeah, I did... I, it, it affected me mentally, physically and emotionally. And it started to manifest as, like, physical illness, too, because my immune system dropped really, like significantly. And I started getting sick more often. Because no sleep, no eating. And so like, I would get colds a lot. I would feel a lot of nausea and throwing up.”* [FD32] | 2 | 2 (7%) |
| **Subcategory: Consequences of last MDE** | | | |  |
| Difficulties taking care of oneself | Any mention of experiencing difficulties taking care of oneself (e.g., showering, brushing teeth, cooking) or maintaining home tasks (e.g., laundry, cleaning) as a consequence of a past MDE. | *“And then, you know, episodes where I don't wash my hair for a week, or I won't brush my teeth, or I'll leave my laundry around like it's just it piles up and it gets to be a lot.”* [FD32] | 15 | 9 (30%) |
| Occupational difficulties | Any mention of experiencing occupational difficulties (e.g., difficulty keeping up with workload, absenteeism, job loss) as a consequence of a past MDE. | *“And then, as it progressed, I didn't really get out of it. And that's when I started getting really, really depressed of, am I ever going to get better because I had to drop out of school, I had to stop working.”* [FD26] | 7 | 7 (23%) |
| Academic challenges | Any mention of academic challenges, difficulties, or consequences (e.g., difficulties keeping up with coursework, dropping out) experienced by participants during a past MDE. | *“So then depression symptoms started and then around six months later, I wasn't going to any of my classes. I wasn't going to my exams. I failed all my classes. And after that I dropped out. I took a semester off after about six months, I dropped out and I spent a semester and then the whole summer not taking classes.”* [FD05] | 18 | 11 (37%) |
| Difficulties functioning socially | Any mention of experiencing difficulties socializing (e.g., withdrawing from others, lack of support, masking depressive symptoms) as a consequence of a past MDE. | *“Um, it was when I was in my second year of high school, so 14. Um, I'm really not sure how it started. But I just started being withdrawn from my friends. Stopped putting in any efforts in my friendships, couldn't talk to people, stopped participating in class when I was someone who participated a lot of class before that. And I just didn't see the point of anything and just kept spiraling.”* [FD05] | 29 | 19 (63%) |
| Strained relationships | Any mention of having strained relationships (e.g., increased conflict, impact on family/children) as a consequence of a past MDE. | *“Well, definitely there is like... There is one, like I was like, very guilty that I hurt him, like he was really devastated. That's just like the number one and of course, other guilt was like, my daughter, like her psychological well-being in a family that's not cohesive. So that was my second guilt.”* [FD11] | 10 | 5 (17%) |
| Engaging in high-risk behaviour | Any mention of engaging in high-risk behaviour (e.g., reckless spending, substance use, risky sexual behaviour) as a consequence of a past MDE. | *“And so my way of coping, I think was to kind of, I guess, make up for that negative experience and engaging in a lot of really destructive sexual behavior as a result. Just not really protecting myself and my health in an effort to I think, yeah, I guess, I guess to make up for the negative experiences. Try to make myself feel better. Yeah, I think I, I would get excessive with, with my alcohol use at that time when I did drink, which is not really often, but when I did, it was excessive and would say things or do things at times that were out of character and not always appropriate. Yeah, might be kind of the worst event, I guess, in addition to just come struggling to start the day get out of bed. And yeah.”* [FD33] | 4 | 4 (13%) |
| **Category: Coping strategies used to cope with past MDEs** | | | |  |
| **Subcategory: Seeking support from others** | | | |  |
| Help from mental health professionals | Any mention of seeking mental health support from professionals (e.g., psychologist, psychiatrist, therapy) to cope with a past MDE. | *“Ah, wow, um, not, not really that I could think of, I had a fantastic therapist. Like, I was seeing a psychologist, and she was, she was fantastic. She knew how to talk to me to get me to like, listen to her in a way that nobody else like, I wouldn't listen to anybody else saying this. And so we had a really good, like relationship in that sense. And she really helped me kind of open my eyes to things and learn so much stuff about myself. So I think that was, I think that was probably honestly, the number one thing that helped me through it was having a good psychologist.”* [FD16] | 37 | 20 (67%) |
| Social support | Any mention of using social support (e.g., having a caregiver, validation, social interaction, talking about it) to cope with a past MDE. | *“It was a small support network. Like, because I didn't want to you know, have everybody know that, oh, I have depression. So it was just a select few. But the select few were very supportive. And because I had pir- prior episodes, so they weren't, it wasn't the first time. So they sort of knew what to do.”* [FD04] | 21 | 12 (40%) |
| **Subcategory: Cognitive coping strategies** | | | |  |
| Cognitive strategies | Any mention of using general cognitive strategies (e.g., challenging thoughts, acceptance, restructuring, diffusion, distraction) to cope with a past MDE. | *“I think, I like, throughout my whole life, I I've always been, maybe, just because it's part of my personality. But I've always been a person who's like, really serious. And really, I don't, I'm not a very optimistic person. So maybe I take things sometimes very personally, or I over exaggerate certain thoughts. But I think having overcome depression, or what depression has sort of led me to experiences is that I can't always think of things that, I can't always think that life is out to get me all the time. And I have to be more open minded. I think being open minded has been one thing that I've improved on in my personal development, and looking at the broader picture.”* [FD04] | 16 | 8 (27%) |
| Developing a more balanced view of the self | Any mention of how developing a more balanced and nuanced view of oneself helped participants cope with a past MDE. | *“And I remember like, I can literally tell you the exact moment that like, my whole perspective of myself, change is watching this video, um, it was a psychologist, and she was talking about people on the autism spectrum that have very special interests. And she was talking about it with such like, interesting, like you could tell, like, she was like, this is so cool that they can do this. And I was like, how is like a stranger, like, so interested and thinks that like, something that I do is so great. And I think it's like despisable and horrible. And it made me really kind of, like, I need to start thinking of myself, and all the things that like, I like, kept, like, hating about myself that like, oh, you're different, or like, I was like, attacking literally everything that like made me me. And it was kind of like a change of perspective. Like, I don't have to hate myself.”* [FD26] | 6 | 4 (13%) |
| Understanding and accepting one's relationship with depression | Any mention of how understanding and accepting one’s relationship with depression helped participants cope with a past MDE. | *“But now, I accept. I think that some days is normal that people feel sad, like other than other days, so at least I think I can differentiate when I'm sad and when I'm, like, depressed and hopeless.”* [FD02] | 6 | 6 (20%) |
| **Subcategory: Behavioural coping strategies** | | | |  |
| Behavioural strategies | Any mention of using behavioural strategies (e.g., activation, self-care, healthier habits, writing, avoidance of triggers) to cope with a past MDE. | *“That would come down to the, you know, eating food that's good for you come down to exercise, which is, like so essential. Trying to get enough sleep, I have some sleep problems unrelated to any of this. And so that's a bit of a challenge, you know, trying to keep stress under control as much as possible, you know, work stress, life stress, whatever. So, yeah, you know, sort of your basic things that people should do, but that a lot of people don't seem to know how to do properly.”* [FD14] | 16 | 11 (37%) |
| Medication | Any mention of using medication to cope with a past MDE. | *“Well, I was prescribed medication immediately. And I so I was prescribed two types of meds, the long term one and the the one to help me like short term, and it like, um, it didn't help right away, I was still, you know, crying all the time. But eventually, a couple of months afterwards, I left because I had, I was finishing my undergrad. So I left and went back home. And then I started feeling slowly started feeling better. So I would say maybe like, a month or two, after I started taking the medication, I started feeling better.”* [FD08] | 15 | 11 (37%) |
| Situational changes | Any mention of how situational changes (e.g., change of environment, reduction in stressors, passage of time) helped participants cope with a past MDE. | *“But by that time, I was kind of assigned to like alternative work and so the stressor was kind of out of my, out of my mind and all that so so it became better in a few months, and I noticed a difference.”* [FD06] | 10 | 7 (23%) |
| **Category: Experiences of being in remission from Major Depressive Disorder (MDD)** | | | |  |
| **Subcategory: MDE symptom improvement** | | | |  |
| Mood improvements | Any mention of participants experiencing mood improvements during remission from MDD. | *“I can literally say I've never been as happy. I still have periods where I'm stressed or sad. But I just feel like, I don't have that continuous, I hate myself voice.”* [FD26] | 22 | 19 (63%) |
| Other depression symptom improvements | Any mention of participants experiencing improvements in depression symptoms (e.g., sleep, energy, concentration) during remission from MDD. | *“I can sleep, but if, I get I get like a good amount of sleep now. I don't sleep the whole day. I can function better. I can go to work. I have I think I have healthy relationships with my friends and family. I can eat. I've been working out, which I didn't do before. So I think I have a healthier lifestyle now. And mentally I think I think I'm the healthiest I've ever been mentally. Yeah. So I'm proud of myself for that. Yeah.”* [FD32] | 4 | 4 (13%) |
| Greater interest and engagement in hobbies and activities | Any mention of participants experiencing greater interest and engagement in hobbies and activities during remission from MDD. | *“I remember like a little bit after coming off of the medication, I just had this huge, like upsurge in this passion and this desire to do all these things that I love, like all of my hobbies, I just wanted to do them all that all at once, and that had kind of been missing for about 10 years. Like yeah, I enjoyed reading, but I didn't love it. And I enjoyed quilting, but I didn't love it. So I, it's almost ironic.”* [FD16] | 4 | 4 (13%) |
| Less rumination, fewer worries, more present | Any mention of participants experiencing less rumination, fewer worries, and feeling more present during remission from MDD. | *“I started worrying quite a bit less about what my friends were thinking of me like, were they hanging out without me? Yeah, pretty normal high school from then on.”* [FD21] | 2 | 2 (7%) |
| **Subcategory: Enhanced identity development and personal growth** | | | |  |
| Greater self-efficacy | Any mention of feeling greater self-efficacy (e.g., more confident, resilient, proud) during remission from MDD. | *“Oh so amazing. Um I feel like once I got out of that relationship, kind of freed myself up that like, I just feel so amazing and so powerful, like a strong woman.”* [FD12] | 20 | 15 (50%) |
| Enhanced identity development and personal growth | Any mention of experiencing identity development and personal growth during remission from MDD. | *“Honestly, I am kind of glad that I went through that because I've grown as a person. And these, well my actions, I mean, very, my depression is very, like, you know, I know the cost and the reason and it was my actions, basically. So it's nothing ambiguous. So I am... I am actually glad that happened and I've grown as a person.”* [FD11] | 15 | 10 (33%) |
| Feeling relieved, free, and/or grateful | Any mention of feeling relieved, free, and/or grateful to be in remission from MDD. | *“It's really, really freeing. It feels way less heavy. It feels just like, a weight lifted off my shoulders. Yeah, it feels it feels really, really good. And I feel. Yeah, I just I feel more of myself again. And I feel inspired. And I feel like excited about things and hopeful about the future. And despite everything, so yeah, feels great.”* [FD24] | 10 | 10 (33%) |
| Experiencing a positive shift in one’s worldview | Any mention of experiencing a shift in worldview (e.g., more optimistic, positive, open-minded) during remission from MDD. | *“And living also more on a, like, you know expanded basis, where I don't look just at what I'm doing today, but also looking forward to something and reporting myself and looking for, think positive things in life rather than thinking of everything as a negative.”* [FD04] | 13 | 9 (30%) |
| **Subcategory: Better functioning** | | | |  |
| Better able to take care of oneself | Any mention of being better able to engage in self-care (e.g., exercising, eating healthier) during remission from MDD. | *“It's been a journey, for sure. But a really, really positive one. I've put a lot of active and healthy habits into my life. And I think that I'm feeling a lot more hopeful about the future, like I've started exercising a lot more, which releases endorphins. And I'm just, I have like more of a healthy habit overall, it's been really amazing. And I think that I'm like really starting to value life a lot more. And just seeing like a really positive outlook. Like, even when we started school this year, it wasn't optimal with online schooling. But I still made the best out of it and made sure I was always checking in on my mental health, and speaking to someone if I ever felt a bit down, so I wouldn't like, go into an episode.”* [FD30] | 8 | 7 (23%) |
| Improvements to academic and occupational functioning | Any mention of participants experiencing improvements to their academic and/or occupational functioning during remission from MDD. | *“Well, at work, I'm much more productive and happy. So, you know, I don't mind going to work. So I work, you know it feels good, and what I really feel that not not, not, not a great relief of say, oh, I found out that environments, like, you know, like everyone, like some people would say, including me, you know, it's a relief to be out of work because, you know, I can go home and relax but it's a that's a different thing from from saying, Oh, I'm relieved to get out of that torture, psychological torture, but I don't have that psychological torture now and homewise yeah, I don't I don't think about work stuff once you know, what gets done that I can concentrate on my, my studies and... Yeah, you know, like a more positive worldview and... positive thinking and all that...”* [FD06] | 4 | 3 (10%) |
| Strengthened relationships and support systems | Any mention of participants experiencing strengthened relationships and/or support systems during remission from MDD. | *“It's been nice. Like, I feel like I'm able to be like, have better relationships. Like I feel like I feel like going away to college was needed, much needed for me. I think I have a better relationships with my family, I, with my, especially my parents. I think my friendships from high school have improved as well. Like I'm much like I might and much and much more sure of myself.”* [FD01] | 6 | 6 (20%) |
| **Subcategory: Residual effects of past MDEs** | | | |  |
| Continuous pressure to manage one’s mental health | Any mention of continuously needing to put effort in to manage one’s mental health (i.e., never feeling fully cured) as a residual effect of a past MDE. | *“Um, I think I even I guess, I hesitate to say I'm no longer depressed because I, I do still take medications to, I guess, maintain this, where I'm at, but it's kind of a constant work in progress and kind of maintenance. And so I'm conscious of the fact that I have to continually and consciously, like deliberately work to, I guess, maintain my mental health and be aware of, of how I'm feeling so that I don't kind of slip back.”* [FD33] | 6 | 4 (13%) |
| Difficulty differentiating “normal” emotions from clinical depression | Any mention of having difficulties differentiating between adaptive/normal emotions from depression symptoms as a residual effect of a past MDE. | *“Um, but it it's hard to find a balance between allowing myself to feel sad and being worried that I might be depressed, you know, but I don't like I kind of know that if um, it's kind of a matter of time. If If my sadness lingers on for more than I don't know, like a, in the month that feels natural to me, then I would get worried and it hasn't happened.”* [FD08] | 6 | 6 (20%) |
| **Section 2: Participants’ experiences with Fears of Depression Recurrence (FoDR)** | | | |  |
| **Category: Presence, frequency, and severity of fears** | | | |  |
| Yes_FoDR | Any mention of participants experiencing FoDR. | *“Yeah, I do worry about the depression coming back.”* [FD06] | 22 | 22 (73%) |
| No_FoDR | Any mention of participants not experiencing FoDR or having FoDR that are not associated with any distress. | *“Um, I would say that I definitely know that that's a possibility. I don't actively sit here and worry about it. Like, it's not sort of preying on me. But it's definitely something that I'm on the lookout for.”* [FD14] | 8 | 8 (27%) |
| FoDR lasts for days | Any mention of participants’ FoDR lasting for days. | *“I think it's just a day. It's a day of thinking about it and saying, Okay, cool. I'm having these thoughts. That's what I'm feeling. And then the next day doing something, that I kind of feel like, okay, screw this. I don't want to be in this zone, so I'll do something that's good for me.”* [FD13] | 5 | 5 (17%) |
| FoDR lasts for hours | Any mention of participants’ FoDR lasting for hours. | *“Yeah, it keeps me up at night. I've had some sleepless nights over it for sure. But I actively work against it. Like I, I write in a journal and I display my thoughts. So it doesn't last more than a couple of hours I, if I'm really feeling scared or anxious, I will speak to someone, or I will check in with myself and I don't want to get to be something really big.”* [FD30] | 7 | 7 (23%) |
| FoDR lasts for minutes | Any mention of participants’ FoDR lasting for minutes. | *“But now, it will last only, like shortest, maybe like 10 minutes, 30 minutes, because I would really try to distract myself from it.”* [FD02] | 13 | 13 (43%) |
| FoDR lasts for seconds | Any mention of participants’ FoDR lasting for seconds. | *“Um... They don't last very long. I mean, they're usually just fleeting thoughts. Um, are usually just fleeting thoughts. If sometimes they do lead to a little crying session, of like maybe half an hour, but then I feel better afterwards or I you know, I just, I just keep tracking.”* [FD24] | 6 | 6 (20%) |
| FoDR lasts for weeks | Any mention of participants’ FoDR lasting for weeks. | *“Yeah, not always weeks and weeks is a bit maybe exag-, like, I would say, one to two weeks.”* [FD08] | 1 | 1 (3%) |
| FoDR lasts for a variable amount of time | Any mention of participants’ FoDR varying in their duration. | *“As long as I'm alone, kind of, you know, when you're sad, and you're like thinking in your own room, like that, that will be present all the time. And once I get the structure, or doing other stuff, it's gone.”* [FD07] | 2 | 2 (7%) |
| FoDR frequency_Few times a year | Any mention of participants experiencing FoDR a few times a year. | *“Not very often, I'd say like, once every few months.”* [FD12] | 8 | 8 (30%) |
| FoDR frequency_Monthly | Any mention of participants experiencing FoDR monthly. | *“I would say it's about once a month.”* [FD30] | 10 | 9 (30%) |
| FoDR frequency_Variable | Any mention of participants experiencing FoDR on a variable basis (e.g., situational, only when triggered, increasing, infrequent, when older). | *“Like every, every once in a while, like it's not regular, but it's just like, if I'm thinking like, okay, what am I gonna, like do in the future with the studies or this job and then I start thinking.”* [FD28] | 10 | 10 (33%) |
| FoDR frequency_Weekly | Any mention of participants experiencing FoDR weekly. | *“Yeah, I would say definitely not daily, but like, maybe weekly.”* [FD36] | 12 | 7 (23%) |
| **Category: Content of fears** | | | |  |
| **Subcategory: Shape of fears** | | | |  |
| An all-encompassing darkness | Any mention of participants’ FoDR resembling feelings of darkness. | *“Yeah. And also, I'd say the darkness like, I just felt like, I guess I felt like, it's a dark cloud of rain it was just always over me. And like, I just felt so like, dark.”* [FD12] | 9 | 6 (20%) |
| Feelings of being trapped | Any mention of participants’ FoDR resembling feelings of being trapped. | *“Hmmm what else? So just being seized in that moment and that place and time. In my mind so it's unpleasant. And not being able to escape that, not being able to escape that outside of the body or get forced to, *inaudible*, forced to experience and feel that.”* [FD06] | 7 | 5 (17%) |
| Specific feelings | Any mention of participants’ FoDR resembling specific feelings. | *“Oh, again, things I said before, like the heaviness, low mood, teariness.”* [FD29] | 6 | 5 (17%) |
| Looming threat | Any mention of participants’ FoDR resembling a looming threat. | *“Yeah feeling down, depressed, worthless, pointless, lonely, claustrophobic, *inaudible*... If I say claustrophobic, claustrophobic and... something bad is gonna happen. Impending doom and stuff like that.”* [FD06] | 5 | 4 (13%) |
| Specific memories of past MDEs | Any mention of participants’ FoDR resembling specific memories from past MDEs. | *“Um, it's just really like, picturing myself like being that low point of my life like three years ago.”* [FD26] | 11 | 10 (33%) |
| Specific thoughts | Any mention of participants’ FoDR resembling specific thoughts. | *“So my psychiatrist, I stopped seeing her, but my psychiatrist in grade 11 that like, was helping me through everything. Once I felt better, she said, you know, you might feel better now, but depression, like once you have it once and even twice, like it's very likely to recur again. So I just want you to be prepared that like, one day in your life, like, even 10 years from now, you can be like, perfect and then like, boom, it can happen again. So, whenever I think of it, I have that I have her voice in my head saying, like, it can come back, it can come back.”* [FD12] | 3 | 3 (10%) |
| **Subcategory: Re-experiencing core MDE symptoms** | | | |  |
| Depressed mood | Any mention of FoDR related to experiencing depressed mood (e.g., low, down, anger/irritability, sad, empty, hopeless, tearful). | *“So something I’ve had sleeping and more, um thinking about self-harming and actually doing it. The thoughts of suicide or actually wanting and having a plan or actually executing it. Well, and just your mood just being very low and just being disengaged from things and everybody else. And even food. Yeah.”* [FD13] | 42 | 21 (70%) |
| Difficulties concentrating, remembering things, or making decisions | Any mention of FoDR related to experiencing difficulties concentrating, remembering things, and/or making decisions. | *“Because I really like missed out so much. Like, I don’t really remember my prom. I don’t remember like my trip to Spain, and like, I just didn’t make any of those memories. So kind of... Yeah, that’s one thing also that I’m that I worry about.”* [FD12] | 6 | 5 (17%) |
| Anhedonia (i.e., diminished interest, pleasure, and/or motivation to engage in activities) | Any mention of FoDR related to experiencing diminished interest or pleasure and/or motivation to engage in activities (i.e., anhedonia). | *“So, like, loss of motivation, and like the ability to do stuff. Increased fatigue, probably is a big worry for me, like brain fog and loss of focus and concentration. Yeah, I guess lots of loss of like, enjoyment in day to day things. And then also, just like, generally, like the increase in negative feelings toward, yeah.”* [FD36] | 26 | 12 (40%) |
| Dissociative episode | Any mention of FoDR related to experiencing a dissociative episode. | *“Um, I guess I’m also afraid of, like, when I do have those thoughts, I’m afraid of the another dissociative episode happening.”* [FD12] | 1 | 1 (3%) |
| Feelings of worthlessness, guilt, and self-criticism | Any mention of FoDR related to feeling worthless, guilty, and/or self-critical. | *“Ah, so start off by saying, like, I'm worthless or I don't deserve to be alive. We, like, I don't deserve the job or I'm like useless at the job, or my family doesn't care about me, or my partner doesn't care about me. And it's just very negative thoughts. So like, it's not even realistic. But it's there, you know?”* [FD13] | 18 | 12 (40%) |
| Comorbid anxiety or panic | Any mention of FoDR related to experiencing anxiety and/or panic. | *“Um, I mean, definitely the panic attacks. Um, but that’s not the first thing I would think of. But that’s a big one that would scare me to have those again. Because it really does feel like you’re, you’re you’re dying. And it’s also like, to a point that it’s like you really have like, zero control over it.”* [FD26] | 2 | 2 (7%) |
| Sleep difficulties | Any mention of FoDR related to experiencing sleep changes and difficulties including fatigue. | *“Probably like a disruption in sleep, disruption in appetite... Just a low mood and like probably fatigue.”* [FD20] | 25 | 17 (57%) |
| Suicidal ideation and non-suicidal self-injury | Any mention of FoDR related to experiencing suicidal ideation, suicide attempts, and engaging in non-suicidal self-injury. | *“Okay, well, obviously, top of the list would be being suicidal again, that would be the first one. The next one down, I guess, would be starting to self-injure again.”* [FD14] | 12 | 8 (27%) |
| Weight and/or appetite changes | Any mention of FoDR related to experiencing appetite and/or weight changes. | *“Yeah, yeah, I'm worried that I'm not going to eat anymore. Because I spent so much time this year trying to gain weight and work out and like, get a, like the achieve good health and the kind of body that I want. And I'm scared that like, if it comes, if it comes, depression comes back, then like all of that work that I did will go down the drain, I'll lose a lot of weight. And I, and yeah, all my work would be wasted. That's what I'm, I'm most concerned of.”* [FD32] | 13 | 9 (30%) |
| Negative cognitions | Any mention of FoDR related to having negative cognitions (e.g., intrusive thoughts, rumination, bleak and pessimistic views of the future). | *“Well, it's honestly, it's scary to think that I was in the place where I couldn't have any kind of positive thought, because I remember. I remember being aware of that. I remember being like, I can't think of anything happy right now. And yeah, that's definitely something that I it's scary to think of that I would ever be in that in that, you know, frame of mind again.”* [FD08] | 18 | 11 (37%) |
| Physical complications and/or pain | Any mention of FoDR related to experiencing physical symptoms (e.g., hair loss, headaches) and/or pain. | *“Okay, symptoms would be getting totally isolated and not being understand by people around you. Being in the, as I say being in the very dark, surrounding. The pain and in the pain, I would mention terrible headaches. But it was, in my case, it was a general pain. But sometime it was terrible headache. And... I will... does also being unable to do things by myself, like not being able to not being able to take care of my cat or not being able to cook?”* [FD22] | 2 | 2 (7%) |
| **Subcategory: Re-experiencing interpersonal consequences or challenges** | | | |  |
| Difficulties socializing | Any mention of FoDR related to experiencing difficulties socializing (e.g., withdrawing, feeling alone/isolated/disconnected, lack of support network). | *“Um, it's always it's also it kind of also has to do with loneliness like I very much picture myself being alone in a dark place. Not being able to see anyone or come close to anyone or you know, grab anyone basically, it's like, depression very much looks isolating to me, because that's how I experienced it.”* [FD08] | 43 | 19 (63%) |
| Negative social evaluation | Any mention of FoDR related to experiencing negative social evaluation and/or feeling like a burden to others. | *“Yeah, I would say that's, that's one part of it. And the other part of it is that if I ever were depressed again, the people around me would be worried about me and that already, you know, I wouldn't want to put anybody through that again.”* [FD14] | 32 | 16 (53%) |
| Strained relationships | Any mention of FoDR related to having strained relationships (e.g., increased conflict, impact on family and/or children). | *“Yeah, for sure, I definitely have feelings of disappointing them. And I also have feelings of hurting them, I don't want to upset them or make them mad or hurt their feelings. Because I definitely can do that. If I if I'm depressed, I can not value their feelings. And I value mine a lot more, which is upsetting.”* [FD30] | 3 | 3 (10%) |
| **Subcategory: Uncertainty about the future and potential impact of another MDE** | | | |  |
| Uncertainty of how another MDE would impact their life | Any mention of FoDR related to being uncertain about how a future MDE would impact one’s life (e.g., falling behind, losing progress, restructuring/adjusting life, not meeting goals, losing control). | *“As soon as I start worrying about losing everything I start thinking if things start going badly again, I'm gonna lose my job, I'm not going to be able to finish this program. And if I don't get good grades this time, I'm not gonna be able to have a second chance like last time, to fix things.”* [FD05] | 37 | 15 (50%) |
| Medication related fears | Any mention of FoDR related to medication changes and decision making (e.g., having to re-take medication, experience withdrawal and side effects, having to take medication forever). | *“Yeah. I also, no there's this one thing I forgot to mention, actually. I, I'm worried about depression returning because I'm worried that it'll come to a point where I would have to go back on antidepressants.”* [FD32] | 5 | 5 (17%) |
| **Subcategory: Occupational and academic consequences** | | | |  |
| Occupational and academic consequences | Any mention of FoDR related to experiencing academic and occupational difficulties (e.g., struggling to keep up with workload, absenteeism, job loss, dropping out of school). | *“Yeah, like just things coming to head. Like, like, currently, I'm just like, I'm like, I'm like, slowly falling behind in school. Like, I can feel it. And I'm like, and falling behind in work as well I feel. So I'm just like, it's gonna come to a point where like, it's all gonna come culminate. And like, I'm gonna, like, I'm gonna fail this semester. And then like, not, not get like, not get like an internship for the summer or, like, you know, my boss will fire me (chuckles).”* [FD01] | 30 | 15 (50%) |
| **Subcategory: Difficulties functioning** | | | |  |
| Difficulties with personal functioning | Any mention of FoDR related to having difficulities maintaining personal functioning (e.g., unable to take care of self and maintain home tasks). | *“Just not enjoying things anymore. Not taking care of myself. Not eating well. Not, not wanting to better myself.”* [FD01] | 24 | 13 (43%) |
| Avoidance | Any mention of FoDR related to becoming avoidant of situations an one’s responsibilities. | *“That was my thing. So I feel like if I ever got into a point that I was kind of trying to sleep more than normal, or trying to use that as an avoidance technique.”* [FD36] | 2 | 2 (7%) |
| **Subcategory: Impediments to personal growth** | | | |  |
| Reduced self-efficacy | Any mention of FoDR related to experiencing reduced self-efficacy (e.g., beliefs that one is unable to cope with difficult situations and/or unable to meet expectations). | *“Yeah. If the worst were to happen, how am I going to cope? How I'm going to deal with it and who's going to come and support me this time? again.”* [FD04] | 14 | 9 (30%) |
| Beliefs about the future of my depression | Any mention of FoDR related to beliefs around the future of one’s depression (e.g., fears around never being cured, concerns about the repeated and ongoing effort to maintain mental health). | *“Yeah, I guess it just like, it doesn't feel effortless at all. And when you put effort into something like you, I guess, like, you hope that it's like to make things better, whereas sometimes it feels like the effort is to just like, avoid something bad happening. If that makes sense.”* [FD36] | 4 | 2 (7%) |
| Diminished sense of self | Any mention of FoDR related to experiencing a diminished sense of self (e.g., loss of purpose, feeling unlike oneself). | *“Um, I would say like, first thing would just be like, I can't go back to that day. It's like, kind of like a fear of like, not wanting to become that person again. So, um, that's why I feel like that's one of the reason like my natural defense mechanism to overcompensate for that is just be like, try to be more happy. Like, do other stuff like don't don't think about it all the time. You might just go back to that loop of always thinking negatively, then thinking like even more negative then it's like thinking more negatively. It's just... Yeah.”* [FD23] | 6 | 5 (17%) |
| **Category: Triggers of fears** | | | |  |
| **Subcategory: Reminders of past MDEs and re-experiencing depression and/or other mental health symptoms** | | | |  |
| Re-experiencing MDE symptoms | Any mention of participants’ FoDR being triggered by re-experiencing MDE symptoms. | *“Being sad. Being angry. Being alone. Um, not being able to concentrate. And maybe not being able to sleep.”* [FD07] | 73 | 25 (83%) |
| Reminders of past MDEs or difficult life experiences | Any mention of participants’ FoDR being triggered by reminders of past MDEs or difficult experiences (e.g., trauma). | *“Yeah, sometimes if I feel really sad or down or, certain thoughts, certain things that happened in the past that are kind of traumatizing, and they can be kind of in the grips of it. It kind of sees us you. And you might even become worse at that moment. And it kind of last kind of a long time in that day so it has happened.”* [FD06] | 40 | 18 (60%) |
| General anxiety | Any mention of participants’ FoDR being triggered by general anxiety. | *“And I think they're very much related. Like, I know that if I were to have anxiety for a very, you know, lot, again, it's about time, you know, if it's consistently anxious, not getting better, I would start, you know, also feeling depressed. But I also worry about depression, regardless of anxiety.”* [FD08] | 6 | 5 (17%) |
| Substance use | Any mention of participants’ FoDR being triggered by substance use. | *“Um so family stress I would say, work stress, past relationships coming up, or that being the main trigger, I would say, um, use of alcohol or any other substances. Um, and what else? A lack of social connection.”* [FD13] | 2 | 2 (7%) |
| **Subcategory: Interpersonal triggers** | | | |  |
| Dealing with interpersonal conflict | Any mention of participants’ FoDR being triggered by experiencing interpersonal conflict (e.g., issues with friends and family, breakups). | *“All the things like breakups. That's a trigger. Or it doesn't even have to be a breakup, you know, like it could just be like someone I had interest in like, not.. Um having like unrequited feelings, I guess. That's like... Um my mood depends on how much sunlight there is. So like, maybe when it gets colder and like less sunlight, like, it could be a trigger.”* [FD03] | 27 | 14 (47%) |
| Feeling alone and unsupported | Any mention of participants’ FoDR being triggered by feeling alone and unsupported. | *“Um, well, the things that kind of triggered my depression in the first place where, you know, being in a bad relationship, having not great friendships, like a lack of support system, being far away from my family, doing badly in school doing badly at work. So I feel like if any of those things started happening again, I might start to feel a little bit depressed, obviously, like, if I lost my job, I would just be heartbroken.”* [FD16] | 20 | 14 (47%) |
| Experiencing grief or loss | Any mention of participants’ FoDR being triggered by grief and/or losing someone. This code captures both actual events and fears about these events potentially occurring in the future. | *“But also, um, well, I, I don't deal very well with grief also. So that's something that I worry about that I worry that if I lose someone very close to me that it could trigger something. Yeah.”* [FD08] | 9 | 6 (20%) |
| Being negatively evaluated by others | Any mention of participants’ FoDR being triggered by being negatively evaluated by others. | *“As I say, it's very much an environment context and either related to my family or related to people around me being criticized, criticized by people around me. You know?”* [FD22] | 2 | 1 (3%) |
| Social comparison | Any mention of participants’ FoDR being triggered by social comparison. | *“Yeah, so it just, I start to compare, and I shouldn't, but I know I shouldn't. But it sometimes I can't help it and I get worried that I am going to be stuck at this job forever. And I won't be like my friends who seem happy and they, like they're getting, they're... They seem like they're at a better point in their life than I am right now. And we're the same age. So when I start comparing, that's when a lot of the worry comes.”* [FD32] | 1 | 1 (3%) |
| **Subcategory: Difficulties functioning at home, work, or school** | | | |  |
| Struggling academically and/or occupationally | Any mention of participants’ FoDR being triggered by academic and/or occupational stressors and challenges (e.g., job loss, struggling in school and at work, unmanageable workload, being unproductive, rejections). | *“Umm there's like, sometimes, sometimes, like, if I have like a meeting with my boss, and, like, like, she'll just give me more work. And then I'll remind me of how much work I do have to do. And like how much more behind I'll be. I get more, I get concerned.”* [FD01] | 33 | 15 (50%) |
| Difficulties functioning | Any mention of participants’ FoDR being triggered by experiencing difficulities functioning (e.g., not taking care of self and home). | *“Um, yeah, just kind of like a decrease in productivity is is a pretty big one. But that kind of, like, encompasses, like, the other ones I was gonna say. Um, so like, like, when you're like, seeing a lack in like hygiene or like normal things that like, you care about just, and that kind of, that's what the problem is. It all comes back to like, feeling overwhelmed.”* [FD26] | 13 | 7 (23%) |
| Feeling overwhelmed and/or stressed | Any mention of participants’ FoDR being triggered by feeling overwhelmed and/or stressed. | *“And, and also stress... I don't know about other people, but for me, for me, it's very personal. Like, I find that when I get very stressed with everything, not really linked to any of the experiences I had during the depression, like it doesn't have to be linked. It just it can be stressed that I'm getting from anything in my life.”* [FD02] | 23 | 14 (47%) |
| Feeling unproductive | Any mention of participants’ FoDR being triggered by feeling unproductive. | *“Oh, yeah. Yeah, that makes sense. I'm just like, if I'm feeling like, sad in general, or if I'm just feeling unproductive. Or if I'm anxious. I'm like, it's just like, not working out. Like in the morning, I wake up sometimes, and I'm like, the amount of things I have to do. And then I'm like, Is it even like... I'm not gonna get it done, what's the point? You know, I feel like that, like, I'm not going to be able to finish everything. And yet another day wasted, something like that.”* [FD01] | 7 | 6 (20%) |
| **Subcategory: Transitions, life changes, and big decisions** | | | |  |
| Going off medication | Any mention of participants’ FoDR being triggered by medication changes (i.e., stopping medication or thinking about stopping). | *“Yeah, because when I stopped taking those antidepressants, I feel like more emotions. Especially like at the beginning when I stopped I felt like a lot more sad. Everything more intense. I feel like when you take antidepressants for me, everything is more like dulled down and like, I'm a bit indifferent, I don't know, less emotional. And then when I stopped, I was like, very emotional and crying a lot for like, maybe two weeks. But I mean, now it's stabilized. But yeah, and then I'm just like, confused. Is this depression? Is this like normal um side effects of just stopping the medication? Or is this just how you're supposed to feel emotions?”* [FD28] | 3 | 3 (10%) |
| Having to make life decisions and future plans | Any mention of participants’ FoDR being triggered by having to make big decisions and plans for my future. | *“Yeah, like a career decision, when I have to make a career decision, that might trigger it or may have triggered the thoughts in the past.”* [FD31] | 12 | 5 (17%) |
| Uncertainty about the future | Any mention of participants’ FoDR being triggered by uncertainty about their futures and the potential impact another MDE could have on their life (e.g., not knowing if/when they’ll get depressed again). | *“I am. I'm, I'm still very worried about that. I feel like it's, um, I mean, right now, I appear to be like, completely fine. Back to normal what it was before, but I feel like, I feel like the fear is the unknown. So you don't know what will it's going to come that might cause it again. Can't really predict that.”* [FD23] | 23 | 12 (40%) |
| Undergoing a transition | Any mention of participants’ FoDR being triggered by undergoing a big transition. | *“It's really mostly transitions and kind of my high expectations for myself and my future, like, right now I'm on the path that I want to be on. But if I don't get to the goals that I want, then maybe it's gonna start getting mad again. So it's transitions and my expectations for myself.”* [FD05] | 10 | 7 (23%) |
| Not meeting expectations | Any mention of participants’ FoDR being triggered by not meeting the expectations of themselves and others. | *“Yeah I have trouble you know changing my goals or settling for what I can *inaudible*, I have certain goals in my mind. So if I don't get to that, I worry about how I'm going to be able to react to it and I'm going to be able to accept that.”* [FD05] | 3 | 2 (7%) |
| **Subcategory: Negative events and environmental factors** | | | |  |
| Environmental factors | Any mention of participants’ FoDR being triggered by environmental factors (e.g., bad living environment, no structure or routine) | *“Okay, yeah. So like, probably when, like, I'm like, in like, a bad financial situation or like, not in a nice living environment, or not around good friends or family or people. Or when I'm, like, more isolated, like, when I'm just having a lot of, like, extra time to not do anything.”* [FD18] | 9 | 7 (23%) |
| Negative life events | Any mention of participants’ FoDR being triggered by negative events (e.g., bad day, accumulation of stressors, new caregiving role). | *“Or, like, if something really bad happens, like, for example, um, my, like, other dog passed away in September. And like, just having that trauma, like, we had to put her down and like, I watched everything and like, I kept having that image in my head of her dying. Um, so that definitely was scary. And like, I was afraid it would return but yeah, but it didn't, thank God.”* [FD12] | 20 | 13 (43%) |
| The COVID-19 pandemic | Any mention of participants’ FoDR being triggered by the COVID-19 pandemic. | *“I think it's just like, at the back of my head. Sometimes I think about that, and especially like, during COVID. When, like, like, the entire society is feeling anxious. And I feel like, maybe, maybe I'll fall back to how I felt before.”* [FD34] | 9 | 6 (20%) |
| **Subcategory: Other triggers of FoDR** | | | |  |
| Experiencing physical sickness and/or body changes | Any mention of participants’ FoDR being triggered by physical sickness and/or body changes. | *“So I would say, I guess it's very personal, but I would say some kind of illness like physical illness and loss. So either, you know, even loss of a pet, not necessarily, you know, any kind of loss or even loss of a job or something, something like that.”* [FD08] | 2 | 2 (7%) |
| Positive experiences | Any mention of participants’ FoDR being triggered by positive experiences. | *“But as soon as I've got out of that, depression, when I started working for the first time, it was ongoing, I kept thinking, what if this is kind of the calm before the storm, everything's gonna go bad again, I'm gonna lose this job.”* [FD05] | 2 | 2 (7%) |
| **Category: Impact of FoDR on daily functioning** | | | |  |
| **Subcategory: A negative impact of FoDR on daily functioning** | | | |  |
| Academic and/or occupational consequences | Any mention of the negative impact of FoDR on academic and occupational functioning. | *“I will waste more time, that's for sure. Like, I will become trapped in a rut and not being able to move on and be productive at that moment, or later on in the day. And sometimes when I when I feel down, I kind of drink coffee or you know, like you wouldn't be motivated to because I work out of home from time to time. Not as much as I would like to, I'm not motivated but when something like that happens, you wouldn't even think about working out.”* [FD06] | 16 | 10 (33%) |
| Engagement in specific behaviours | Any mention of the negative impact of FoDR on specific behaviours. | *“Um, I think like, they definitely have made me avoid certain things that I worry might trigger my depression coming back.”* [FD36] | 15 | 6 (20%) |
| Increased anxiety | Any mention of how FoDR led to increased anxiety, panic, or worry. | *“Umm, it has a lot of impact. Like, it can be just light, as just make you worry, like, just being worried is not something you want. Think you.. you don't want to be constantly worried about something.”* [FD02] | 23 | 17 (57%) |
| Influenced future decision making and choices | Any mention of how FoDR had a negative impact on one’s ability to make decisions about their future and act or behave a certain way (e.g., struggles to make decisions, choose life goals, and avoidance of high-risk situations, personality changes). | *“I definitely don't do the same things I used to before. Like, I'm not the same person, it's been eight years. But I do feel like there was a shift in my personality from before and after my depression, like, I'm a lot less of a risk taker. I'm a lot less, you know, worry-free, I am more careful, I'm more aware, sometimes in an annoying way, you know, I don't want to be that careful and aware.* [FD08] | 13 | 5 (17%) |
| Influences medication decision making | Any mention of the negative impact of FoDR on medication decision making. | *“Um, I guess maybe just didn't decision to not go off the antidepressants. Like, if I'd really been like, completely like, Oh, it's not coming back. Maybe I would have listened to someone and gone off of them. But I just I didn't want to go through the possibility or even like the if I was totally fine the withdrawal?‬”* [FD21]‬‬‬‬‬‬‬‬‬‬‬‬‬‬‬‬‬‬‬‬‬‬‬‬‬‬‬‬‬‬‬‬‬‬‬‬‬‬‬‬‬‬‬‬‬‬‬‬‬‬‬‬ | 6 | 3 (10%) |
| Negative impact on relationships | Any mention of how FoDR had a negative impact on one’s relationships (e.g., withdrawal, reduced relationship quality). | *“Yeah, like, maybe not going into a relationship. Because I'm scared like, someone's going to have to, like, deal with me and my like, downs. Like if my depression comes back then... Yeah. I don't want someone to like have to deal with that.”* [FD28] | 14 | 9 (30%) |
| Negative mood changes | Any mention of how FoDR negatively impacted one’s mood. | *“Um, I guess I just feel sad or, like, more overwhelmed, but also disheartened. Yeah. Yeah. Yeah.”* [FD01] | 21 | 14 (47%) |
| Not taking care of self and home | Any mention of the negative impact of FoDR on one’s ability to function and take care of themselves and/or their home. | *“Yeah. My plants dying on me, like not, not, spending way too much money. Um not cleaning my apartment, just letting it letting it fall to a state. And then I have to clean it all up again. And then it just goes back into that state. Having to sleep too much, which makes me not want to not have enough time to work. And if I don't have enough time to work, I'm not working. I'm not getting my work done. falling behind. Yeah, that kind of stuff.”* [FD01] | 4 | 2 (7%) |
| Reduced interest and/or engagement in activities | Any mention of how FoDR negative impacted one’s interest and engagement in activities. | *“Yeah, I definitely don't feel like doing anything. And that includes stuff that like I like to do. Either like, yeah, like. Like, I'm like, like, watching watching a TV show is not as fun as anymore. Like, just like, I'll watch it. It'll distract me for a bit, then I just have to go back.”* [FD01] | 8 | 4 (13%) |
| Increased self-doubt | Any mention of how FoDR led to increased self-doubt. | *“Um, good question. I mean, because it impacts me for such a short period of time, usually that it's hard, but I say it impacts my thoughts about myself. My, sort of makes me doubt my coping skills. Makes me doubt my, my strength. Yeah, I guess it's mostly mostly thoughts, yeah, that it impacts.”* [FD24] | 2 | 1 (3%) |
| Sleep difficulties | Any mention of how FoDR negatively impacted one’s sleep. | *“Yeah, it keeps me up at night. I've had some sleepless nights over it for sure. But I actively work against it. Like I, I write in a journal and I display my thoughts. So it doesn't last more than a couple of hours I, if I'm really feeling scared or anxious, I will speak to someone, or I will check in with myself and I don't want to get to be something really big.”* [FD30] | 5 | 3 (10%) |
| Snowball effect | Any mention of how FoDR had a “snowball effect” where FoDR led to greater stress, and increased hypervigilance to symptom and mood changes. | *“I guess it would. Yeah, it just like, I feel like it's more of a snowball effect. You know, one thing leads to another leads to another and you're not able to just do anything. Yeah, yeah, I guess.”* [FD01] | 13 | 11 (37%) |
| **Subcategory: A positive impact of FoDR on daily functioning** | | | |  |
| Greater engagement in health behaviours | Any mention of how FoDR led to greater engagement in positive health behaviours (e.g., recognizing warning signs and proactively addressing them, seeking support). | *“For me, it made me more aware of the fact that I need to do more coping, like engage more coping strategies, practice it, and actually monitor them over time and see what works for me.”* [FD13] | 28 | 12 (40%) |
| Positive personality changes and growth | Any mention of how FoDR led to positive personality changes including personal growth and knowing oneself better. | *“Also, it's made me more present in a way of knowing where I'm at emotionally or mentally. Ah, so I would say in a way, it's kind of made me emotionally stronger, which is weird. Like, it's very weird, but like, in a way it has, because I've seen where don't want to be.”* [FD13] | 17 | 9 (30%) |
| Relationship improvements | Any mention of how FoDR led to relationship improvements (e.g., improved relationship quality, better communication). | *“Um, it's made me push myself more and try new things, and strengthened my relationships. And also, it's made me realize what I deserve and what I don't deserve, I guess. Like, if anyone ever treats me the way that my ex treated me, I'd be like, no, get out. Like I deserved more than you. So.”* [FD12] | 5 | 3 (10%) |
| **Subcategory: No or little impact of FoDR on daily functioning** | | | |  |
| No or little impact on daily functioning | Any mention of how FoDR have little to no impact on one’s life and ability to function. | *“But I would worry. Maybe a few days. But now is like, I don't worry as much. The worry doesn't last that long. So definitely no impact on my life. And emotionally too. It doesn't. I wouldn't say it affect that much.”* [FD02] | 28 | 15 (50%) |
| **Category: Coping with FoDR** | | | |  |
| **Subcategory: Cognitive strategies** | | | |  |
| Cognitive strategies | Any mention of using general cognitive strategies (e.g., challenging thoughts, acceptance, thought restructuring, diffusion, distraction) to cope with FoDR. | *“Because I know, instead of digging into the memories, I can just stop there. And then this sort of distract myself, think about something else, I have better things to do than this.”* [FD02] | 79 | 29 (97%) |
| Shifting perspective to be more positive and open | Any mention of participants coping with their FoDR by shifting their perspective to be more positive and open. | *“I'm not sure. Um, not sure. Instead of feeling worried most of the time, um like, I feel grateful that I'm not how I used to be. That's, that's the that's the thought I have most of the time, actually, other than just worrying about how it might come back. I think about how I'm great at the moment, and I feel happy about that. Maybe that helps me not think, not worry that it will come back.”* [FD31] | 23 | 15 (50%) |
| **Subcategory: Seeking social support (e.g., friends, family, mental health professional)** | | | |  |
| Social support | Any mention of using social support (e.g., having a caregiver, validation, social interaction, talking about it) to cope with FoDR. | *“Um, I think having a really big support at home is extremely effective.”* [FD07] | 41 | 20 (67%) |
| Help from mental health professionals | Any mention of receiving support from a mental health professional (e.g., psychologist, psychiatrist, therapy) to cope with FoDR. | *“Well I'm continuing to take my medication at at a lower dose than originally. And I continue to see a psychiatrist to manage that medication. I, yeah, mainly just kind of taking care of my mental health generally has been my strategy.”* [FD33] | 15 | 10 (33%) |
| **Subcategory: Behavioural strategies** | | | |  |
| Emotion focused coping strategies | Any mention of using emotion-focused strategies (e.g., self-soothing, emotion regulation) to cope with FoDR. | *“I don't have a strategy. I just, I think about it coming back. I know, I can't talk myself out of it, for example. So I don't try that. It's just understanding that it's normal and that I already went through it. Um not living my life and feeling my emotions should be enough for it not to come back.”* [FD07] | 6 | 6 (20%) |
| Participating in mastery-oriented activities | Any mention of engaging in mastery-oriented activities (e.g., learning something new, working, being productive) to cope with FoDR. | *“Mm hmm. I think like speaking, like, reaching out to my friends more, um playing with my dog helps a lot. And also like, learning new hobbies and just, yeah, learning things.”* [FD28] | 10 | 7 (23%) |
| Engaging in health behaviours | Any mention of engaging in positive health behaviours (e.g., exercise, listening to music, self-care) to cope with FoDR. | *“Definitely like when I was first, very depressed, and I went to see like, a psychiatrist, like, one of the advice he gave me was like to exercise more and things like that. Try to like whenever those thoughts come back, I just tend to go exercise.”* [FD23] | 35 | 18 (60%) |
| Directly addressing sources of FoDR | Any mention of using problem solving to direct addressing sources of FoDR (e.g., reduce stressors, change environment) as a way of coping with FoDR. | *“My worries are usually centered on something that's changed. So I try to see how I can change that back. So like I was saying, if I start sleeping, less, or this, start working too many hours in it, so then, then I don't eat as much and I become more tired, I started noticing that. So then I try to go back to the structure.”* [FD05] | 14 | 10 (33%) |
| Relaxation strategies | Any mention of using relaxation strategies to cope with FoDR. | *“I do yoga as well, and meditations. It helps as well.”* [FD23] | 15 | 11 (37%) |
| **Subcategory: Personal growth and development** | | | |  |
| Developing a more balanced and/or positive view of the self | Any mention of how developing a more balanced or positive view of oneself is a useful coping strategy for FoDR. | *“It's really just... I guess the best thing would be to learn how to be comfortable being by yourself. I mean, it depends on like, why people are depressed. But for me, it was also like, tied to like, loneliness and like, I guess rejection. So for me, it was like, okay, learning to like, build a better relationship with yourself. And just like, time, I guess it's, for me, it's really about time, just plenty of time pass and you will feel the wounds.”* [FD03] | 27 | 11 (37%) |
| Understanding and accepting one’s relationship with depression | Any mention of how understanding and accepting one’s relationship with depression is a useful coping strategy for FoDR. | *“But I, I think I'm much stronger than I was when I first had depression, because first of all, I'm much more aware of what it is. And also my family's aware of it, like, you know, my friends are aware, I know who to talk to, of my friends, if I have to. So, and I know, also I know what to say to a doctor, or you know, how to deal with it, who to go to...”* [FD08] | 37 | 18 (60%) |
| **Category: Situations that reduce FoDR (protective factors)** | | | | |
| Feeling confident about one’s coping skills | Any mention of experiencing a reduction in FoDR due to feeling confident in one’s ability to cope. | *“My coping strategies mainly.”* [FD04] | 3 | 3 (10%) |
| Distraction | Any mention of experiencing a reduction in FoDR when distracted (e.g., on vacation, engaging in hobbies, cooking, cleaning) | *“I guess, distractions from the people that I talked to, from my close friends. And so that that would kind of distract me away, like, we would talk about other stuff. Even distracting me from the current pandemic and whatnot.‬”* [FD06]‬‬‬‬‬‬‬‬‬‬‬‬‬‬‬‬‬‬‬‬‬‬‬‬‬‬‬‬‬‬‬‬‬‬‬‬‬‬‬‬‬‬‬‬‬‬‬‬‬‬‬‬ | 8 | 7 (23%) |
| Feeling competent, productive, and accomplished | Any mention of experiencing a reduction in FoDR due to feeling competent, productive, and accomplished. | *“When people point out progress that I've made recently, when, when I was at work for one year, my boss had kind of an evaluation of my work so far, and he was telling me when you started you were very quiet, very shy, not unsure of what you were doing. Now you seem more confident in your work and, and hearing people say that, people who don't know anything about my past point out the progress I've made made me make me think that maybe I've learned enough by myself, maybe I'm less negative now that I have a better outlook on life. And maybe that won't happen again, the depression won't happened again.‬”* [FD05]‬‬‬‬‬‬‬‬‬‬‬‬‬‬‬‬‬‬‬‬‬‬‬‬‬‬‬‬‬‬‬‬‬‬‬‬‬‬‬‬‬‬‬‬‬‬‬‬‬‬‬‬ | 9 | 8 (27%) |
| Situational improvements | Any mention of experiencing a reduction in FoDR due to positive changes happening in one’s life and/or feeling that things are going well. | *“And then it's very recently that like, it's like I think it's like a new phase of my life. Like I got a job and then I have like a whole new thing going on. So with this, with these and everything, I get busier and I have something else... I have goal in my life now. So I have weightless of them.‬”* [FD02]‬‬‬‬‬‬‬‬‬‬‬‬‬‬‬‬‬‬‬‬‬‬‬‬‬‬‬‬‬‬‬‬‬‬‬‬‬‬‬‬‬‬‬‬‬‬‬‬‬‬‬‬ | 5 | 4 (13%) |
| Mood improvements | Any mention of experiencing a reduction in FoDR due to positive mood changes (e.g., feeling happy, positive, focused). | *“When I'm happy, when I'm with friends that I like, when I'm with my girlfriends. Yeah, when I'm just quite happy. Not worrying about the future or not, like when I'm just happy at the moment, I don't think about depression coming back.‬”* [FD31]‬‬‬‬‬‬‬‬‬‬‬‬‬‬‬‬‬‬‬‬‬‬‬‬‬‬‬‬‬‬‬‬‬‬‬‬‬‬‬‬‬‬‬‬‬‬‬‬‬‬‬‬ | 8 | 8 (27%) |
| Positive social interaction and/or feeling supported | Any mention of experiencing a reduction in FoDR due to having positive social interactions and feeling supported by others. | *“Being around my friends, my family, my new puppy, um, I'm in such a happy, healthy relationship right now. So when I'm with him.‬”* [FD12]‬‬‬‬‬‬‬‬‬‬‬‬‬‬‬‬‬‬‬‬‬‬‬‬‬‬‬‬‬‬‬‬‬‬‬‬‬‬‬‬‬‬‬‬‬‬‬‬‬‬‬‬ | 8 | 8 (27%) |
| **Section 3: Influence of the COVID-19 pandemic on FoDR (not reported in text)** | | | |  |
| **Category: Impact of the COVID-19 pandemic on FoDR** | | | |  |
| **Subcategory: Negative impact of COVID-19 on FoDR** | | | |  |
| Impact on job security, productivity, work, and future goals | Any mention of how FoDR were influenced by the negative impact that the COVID-19 pandemic had on job security, productivity, work, and future goals. | *“Yeah. I guess another thing I can you talk about is, I guess there's another stress-, I guess, a stressor like, like, right now I could I kind of deroute like a pandemic kind of deroute of my, my career plans as well. So that didn't sit well to me either. Like I wanted to volunteer at a certain place, and that didn't get to happen.”* [FD06] | 8 | 7 (23%) |
| Increase in negative emotions | Any mention of how FoDR were influenced by the negative impact that the COVID-19 pandemic had on emotions (e.g., increased fear, stress, frustration, and guilt). | *“Yeah, before I left I was really sad. But I actually I'm not sure it was because of the COVID *chuckles*. I'm not sure. Like it was related to COVID. But like, not being able to, like go physically to school. And I feel like it brings a lot of symptoms, like low motivation. So yeah, I think. Yeah, I mean, now that I think about it, maybe I might be a bit scared that *chuckles*, that it's gonna bring back some of the symptoms of depression.”* [FD28] | 12 | 8 (27%) |
| Increase in negative thoughts | Any mention of how FoDR were influenced by the negative impact that the COVID-19 pandemic had on one’s thoughts (e.g., increased anxiety, worrying, uncertainty). | *“Like not knowing, where the future is going to go? Like of the uncertainties like not very reassuring. Like I have always worked a lot and not being able to work.... Like working can be like a coping mechanism I guess. Like keep yourself busy. And like, when you don't have that, it's like hard to bounce back as fast. Just like meeting people is hard too.”* [FD03] | 18 | 14 (47%) |
| Loss of interest and engagement in activities | Any mention of how FoDR were influenced by the negative impact that the COVID-19 pandemic had on one’s interest in and ability to engage in activities. | *“Staying from home, and like, I don't really like I also feel like I used to really, like, enjoy going out. I don't really go out anymore.”* [FD01] | 17 | 10 (33%) |
| Loss of structure and routine | Any mention of how FoDR were influenced by the negative impact that the COVID-19 pandemic had on people’s ability to maintain structure and routine in their lives. | *“Yeah, yeah, I definitely feel like, you know, like life before I had a structure, I had a routine. Like, I wouldn't really have to motivate myself as much. And yeah, like, I used to, like, no, like, Oh, this is work time. Oh, this is cool time. Or this, this, you know, like, I can spend enjoy my time here. Like, here. It's just, it's all just one big block of time. So it's just like, yeah.”* [FD01] | 17 | 12 (40%) |
| Social consequences | Any mention of how FoDR were influenced by the negative impact that the COVID-19 pandemic had on one’s social functioning (e.g., increased social anxiety, harder to meet people, social isolation/loneliness). | *“Yeah, so I think socially, it's definitely affected my worries about being isolated. And like, not really having a good support system if I were to have like depression return”* [FD36] | 15 | 14 (47%) |
| **Subcategory: No or little impact of the COVID-19 pandemic on FoDR** | | | |  |
| No or little impact of COVID-19 on FoDR | Any mention of the COVID-19 pandemic having little to no impact on participants’ FoDR. | *“Um, actually, no, not... Actually no just because like I have a very good relationship with my husband and my daughter. We have a housekeeper, a nanny who comes all the time. So there's was still some other person in the house for my daughter to interact with during the first lockdown.”* [FD11] | 15 | 15 (50%) |
| **Subcategory: Positive impact of the COVID-19 pandemic on FoDR** | | | |  |
| Positive impact of COVID-19 on FoDR | Any mention of how FoDR were influenced by the positive impact that COVID-19 had on participants' lives. | *“Um, yeah, I guess, I've been, like, I've been just really busy with COVID. I had, like, when I was working full time, I was really busy working full time. And like, to be honest, the COVID situation has kind of been somewhat better for me than for other people. Because I get to kind of be with people I'm close with, but also be on my own because I'm more introverted.”* [FD20] | 6 | 6 (20%) |
